# Supplementary material for: Expansion microscopy of neutrophil nuclear structure and extracellular traps
Source: Biophys Rep (N Y). 2022 Dec 10;3(1):100091. doi: 10.1016/j.bpr.2022.100091 (PMC9813678; doi:10.1016/j.bpr.2022.100091)
Supplement: Document S1. Figures S1–S12 [file mmc1.pdf]

Biophysical Reports, Volume *3*

Supplemental information

Expansion microscopy of neutrophil nuclear structure and extracellular traps

Jason Scott Holsapple, Lena Schnitzler, Louisa Rusch, Tobias Horst Baldeweg, Elsa Neubert, Sebastian Kruss, and Luise Erpenbeck

Supporting material

Expansion microscopy of neutrophil nuclear structure and extracellular traps

Jason Scott Holsapple[1*], Lena Schnitzler[2*], Louisa Rusch[3], Tobias Horst Baldeweg[3], Elsa Neubert[4], Sebastian Kruss[2,4,5+] and Luise Erpenbeck[1+]

1 Department of Dermatology, University Hospital Münster, Von-Esmarch-Strasse 58, 48149 Münster, Germany

2 Department of Chemistry, Ruhr-University Bochum, Universitätsstrasse 150, 44801 Bochum, Germany

3 Department of Dermatology, University Medical Center Göttingen, Robert-Koch-Strasse 40, 37075 Göttingen, Germany

4 Fraunhofer Institute for Microelectronic Circuits and Systems, Finkenstrasse 61, 47057 Duisburg, Germany

5 Center for Nanointegration Duisburg-Essen (CENIDE), Carl-Benz-Strasse 199, 47057 Duisburg

* Equal contribution

Corresponding authors: luise.erpenbeck@ukmuenster.de, sebastian.kruss@rub.de

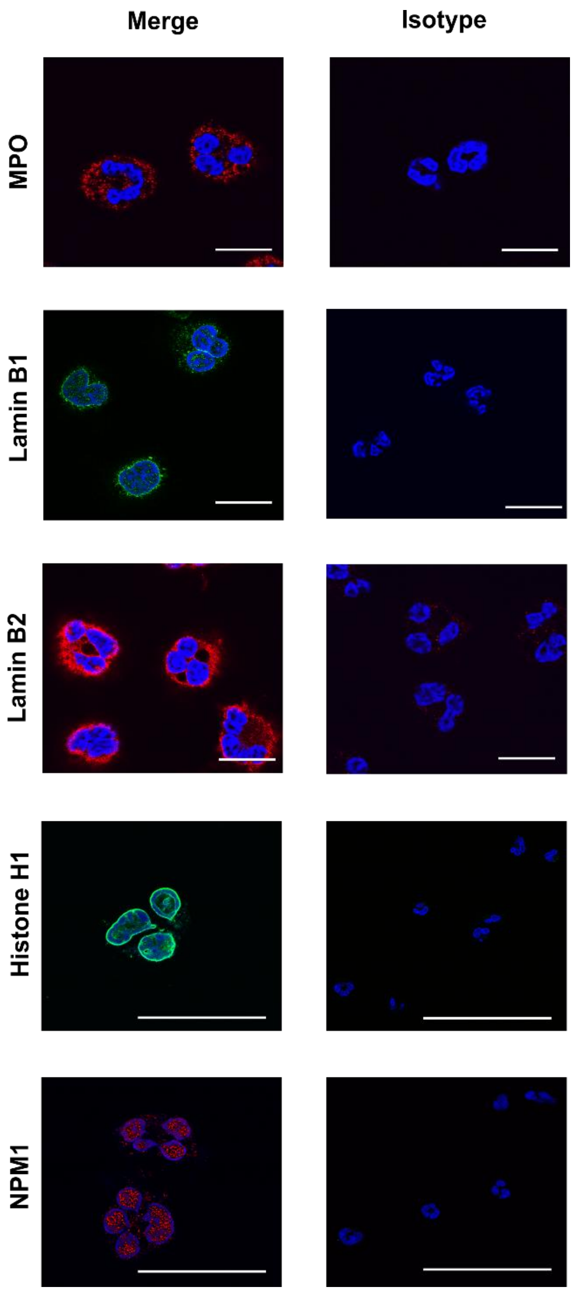

Supplementary Figure 1: Isotype controls for immunofluorescence staining of neutrophil structures, including cytoplasmic and nuclear proteins*: Myeloperoxidase (MPO, first row) as a cytoplasmic protein, Lamin B1 (second row) and Lamin B2 (third row) as components of the nuclear lamina of neutrophils, and histone H1 (fourth row) and nucleophosmin (NPM1, fifth row) as nuclear proteins. Column 1 shows expanded chromatin and stained structures merged. Column 2 shows the respective isotype controls for these structures. Scale bars = 50 µm.*

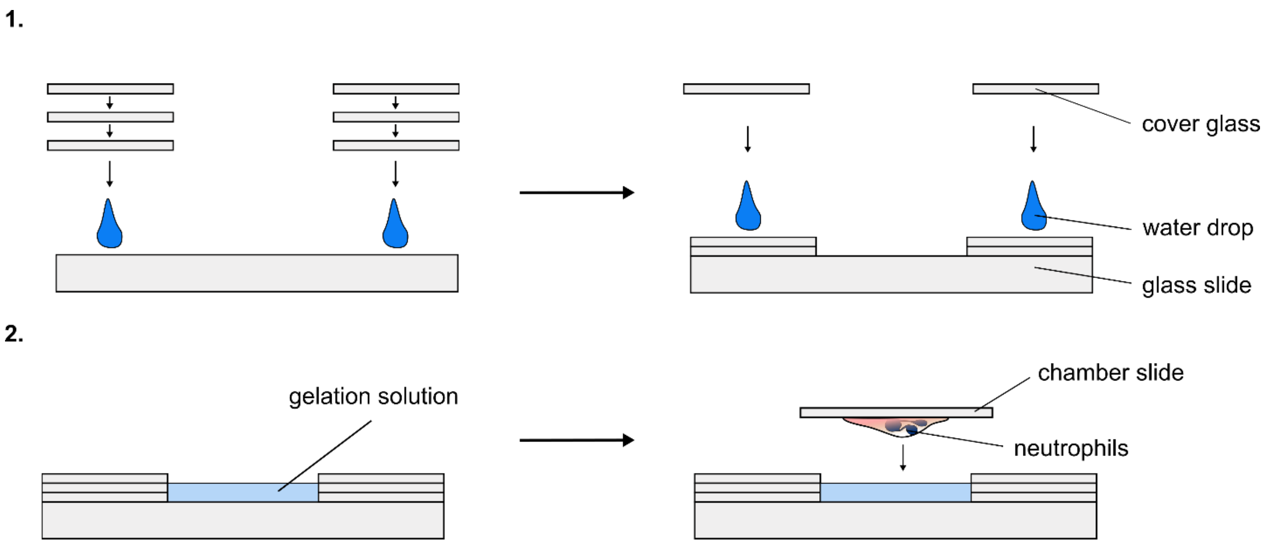

Supplementary Figure 2: Schematic of gelation chamber assembly*. As outlined in the methods, a gelation chamber was assembled as an alternative to the 8-well chamber slides. Cover glass was attached to a glass slide using water drops, with a drop of water in between each layer of glass. The gelation solution was then introduced to the newly formed chamber and chamber slides with stained and labeled neutrophils were carefully lowered into the gelation solution.*

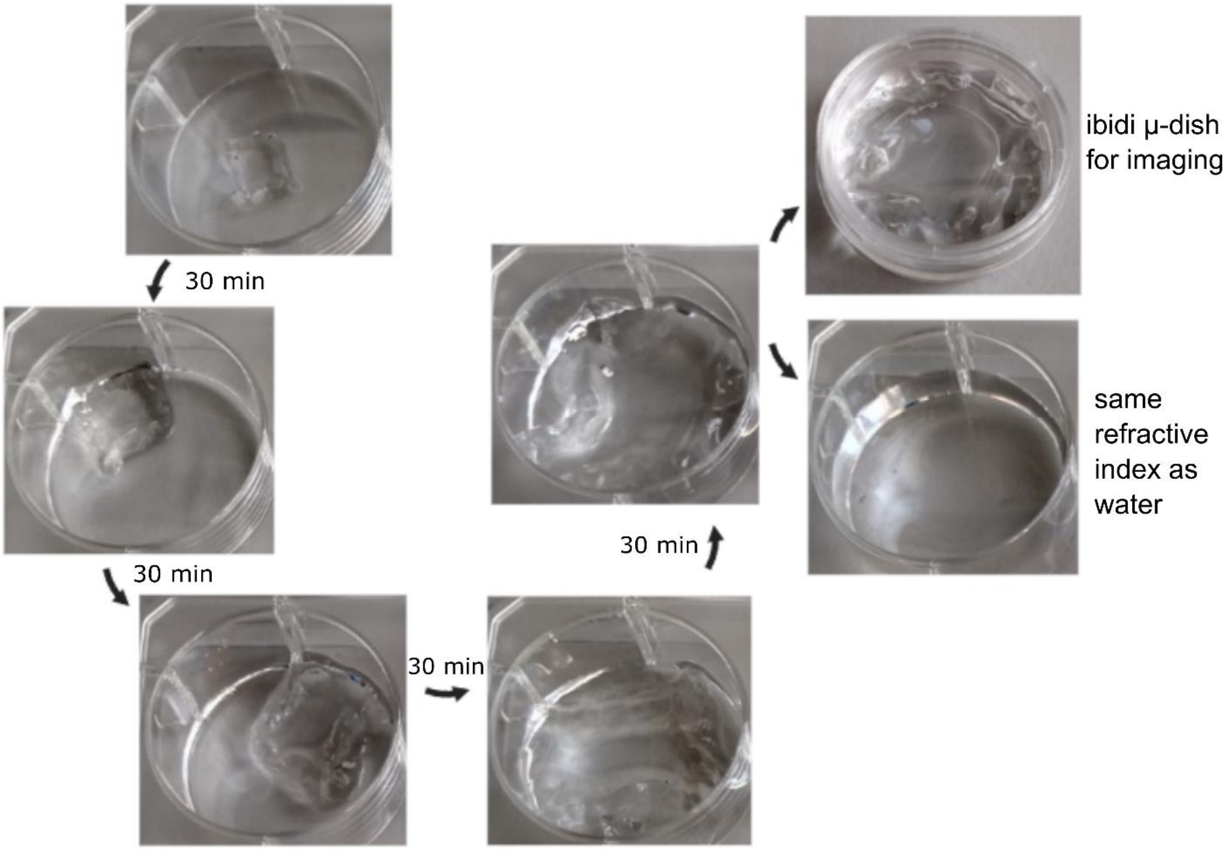

Supplementary Figure 3: Photographs of expansion time series. *Image series showing expansion of gel following addition and exchange of distilled water (30 minutes between each, pictures taken during water exchanges). An ibidi µ-dish was used for imaging, facilitating simple gel handling with higher objectives. Final image shows expanded gel in water, demonstrating the gel having matched the refractive index of water. Image created in BioRender.com.*

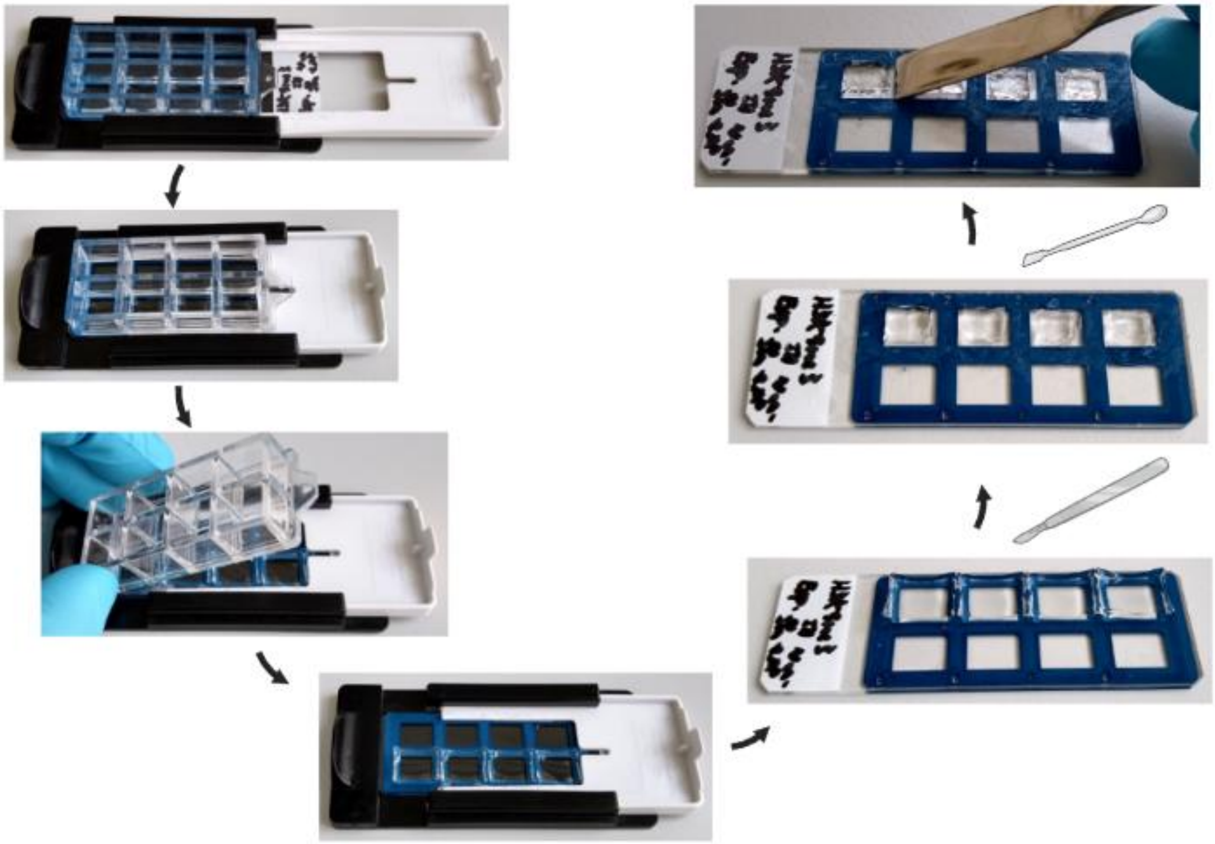

Supplementary Figure 4: Photographs of expansion microscopy technique (8-well format): *Example images of gel processing and removal from 8-well chamber slide (Nunc Lab Tek II). Following chamber removal, gel edges were removed using a disposable scalpel and then gels removed from the slide using a flat spatula. Image created in BioRender.com.*

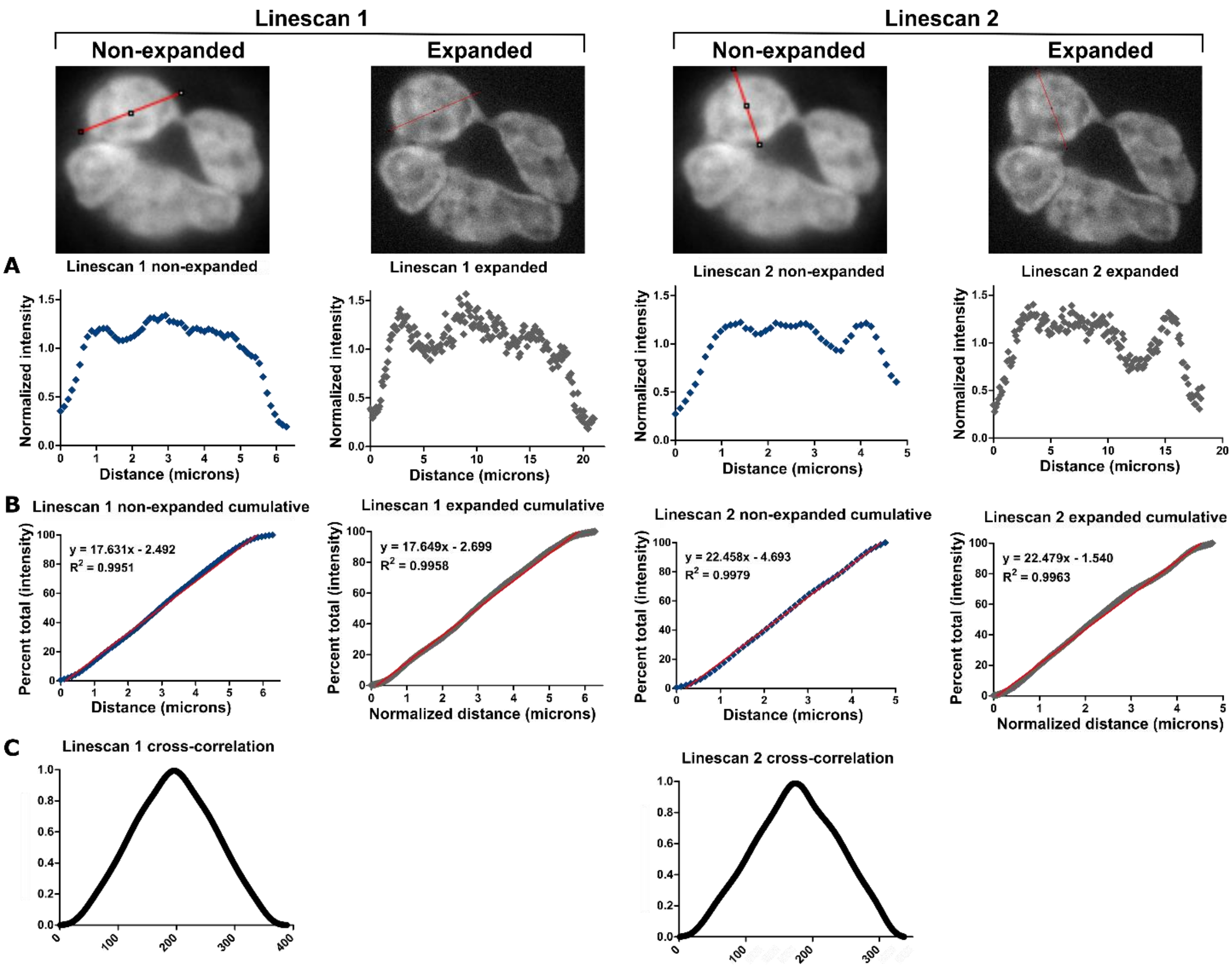

Supplementary Figure 5: Analysis of chromatin distribution pre- and post-expansion. *The distribution of chromatin in the same cell pre- and post-expansion was analyzed. The normalized intensity (gray value divided by mean gray value) corresponding to each line scan is shown in* ***A****. Cumulative plots representing the change in distribution of chromatin over the line scan are shown in* ***B****. Distance was normalized to the non-expanded range so the values could be compared, and a linear regression was performed. The high degree of similarity between the non-expanded and expanded slopes suggests similar distributions. Cross-correlation analyses of line scans from the non-expanded vs expanded cells are shown in* ***C,*** *and were performed using MATLAB (version R2019b) after data interpolation (linear method, 'interp1' function). The peak suggests a high degree of cross-correlation between the interpolated non-expanded and expanded line scans.*

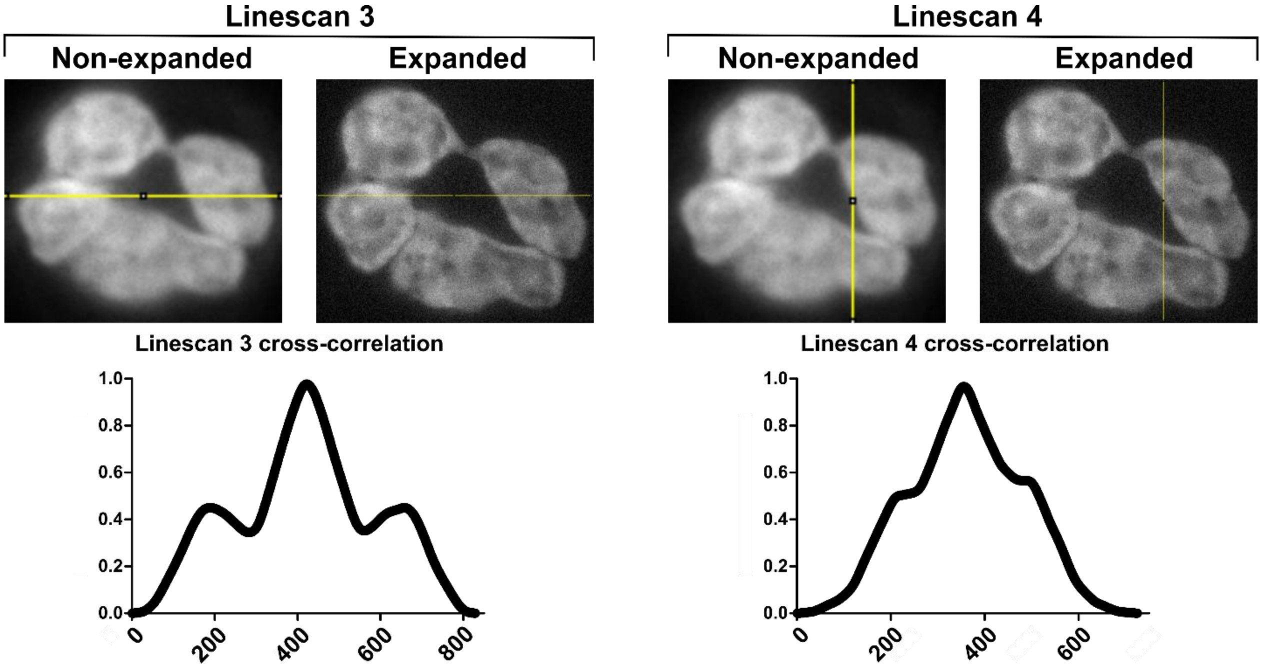

Supplementary Figure 6: Cross-correlation analyses of whole-image line scans*.* *Additional line scans were subjected to cross-correlation analysis as in Figure 4. Whole image line scans were analyzed to further demonstrate the high degree of correlation between the same areas of the same cell pre- and post-expansion.*

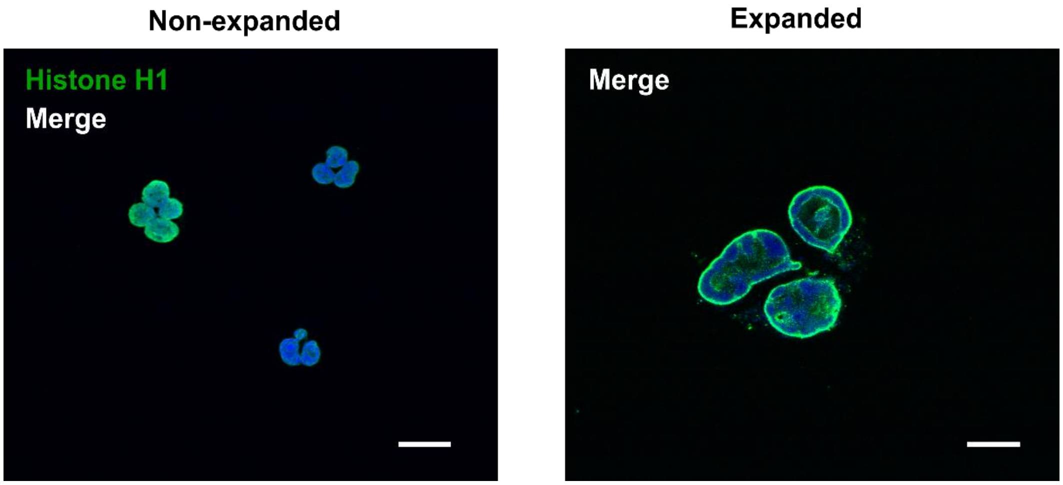

Supplementary Figure 7: Expansion microscopy and immunofluorescence staining of neutrophil nuclear border proteins*. Histone H1 was stained as a nuclear protein involved in unique higher-order structural conformations. Left shows non-expanded cells and right shows expanded cells. Scale bars = 10 µm.*

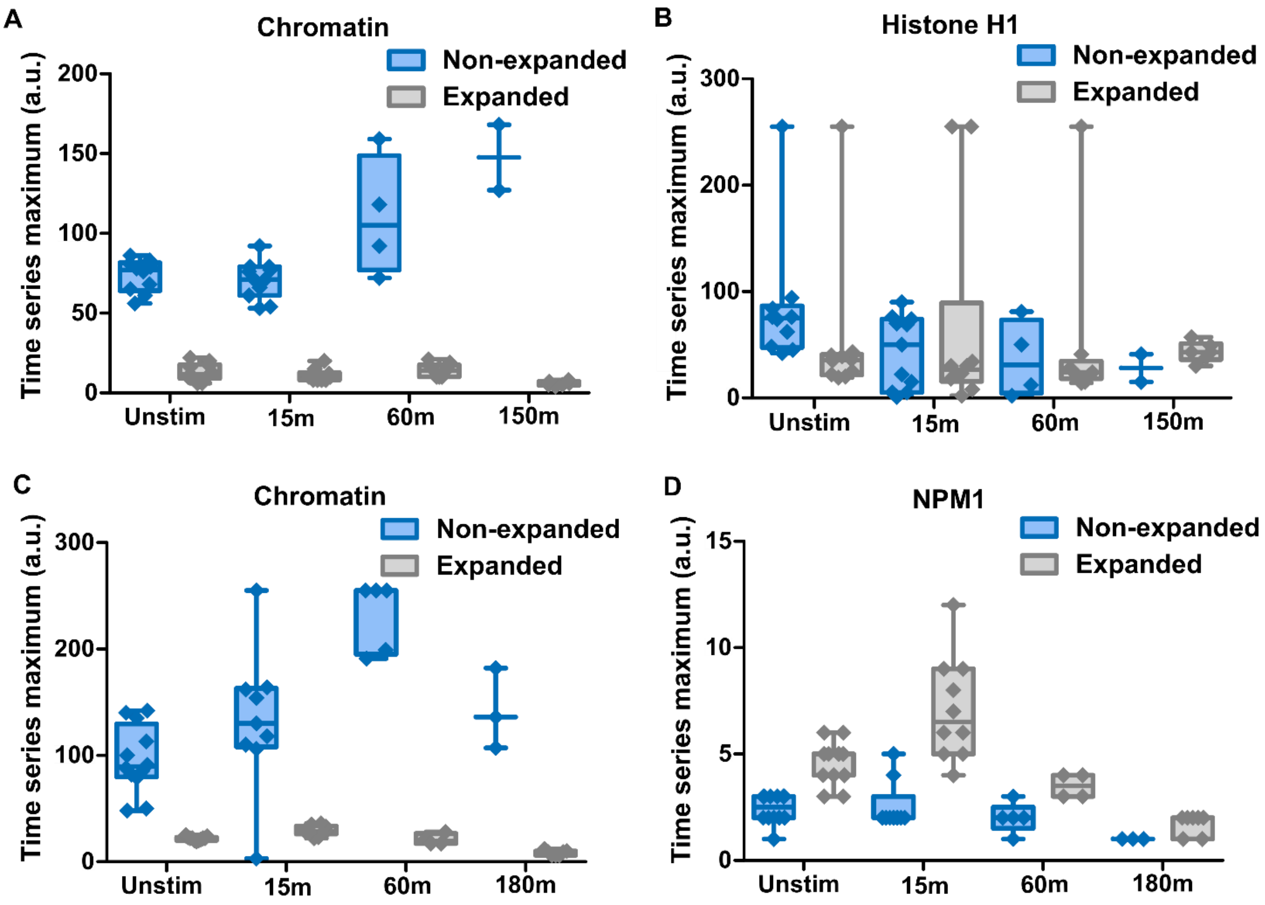

Supplementary Figure 8: Maximum intensity of paired chromatin and histone H1 or NPM1 in a time series*: Boxplots of the maximum intensity for chromatin and histone H1 (**A**, **B**) and chromatin and NPM1 (**C**, **D**). Plots are paired and respectively labelled as non-expanded and expanded for each. Whiskers represent the maximum and minimum for each boxplot.*

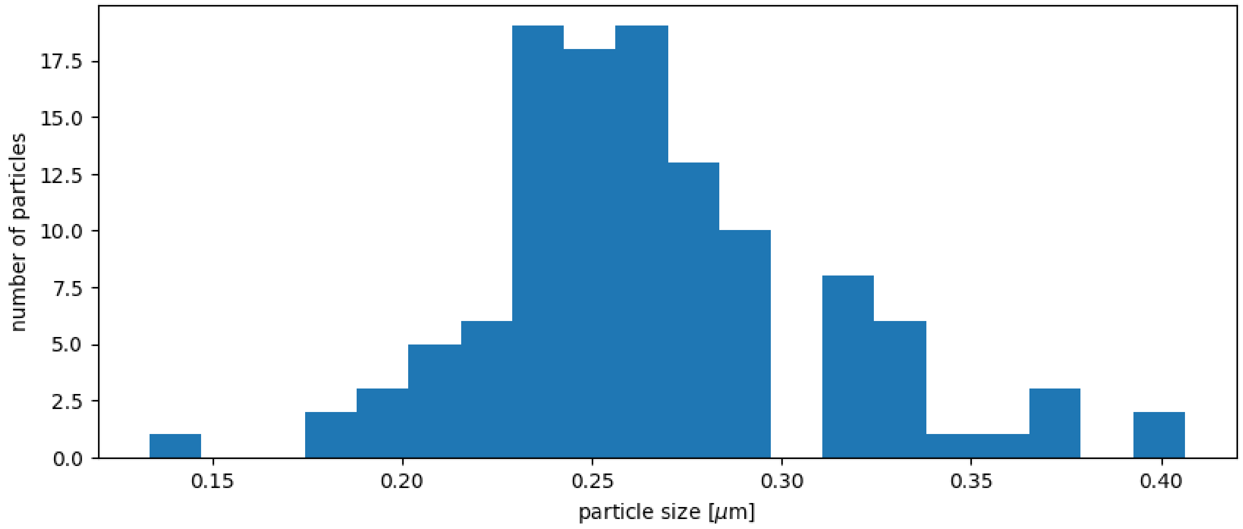

Supplementary Figure 9: Histogram of granular particle diameters*: The granular particle diameter of the histone H1 staining of a neutrophil after 150min is shown. The mean diameter is 266nm ± 45nm which corresponds to 54.29 nm ± 9.18 nm before expansion. The small granular structures are without expansion microscopy below the resolution limit and would not be visible.*

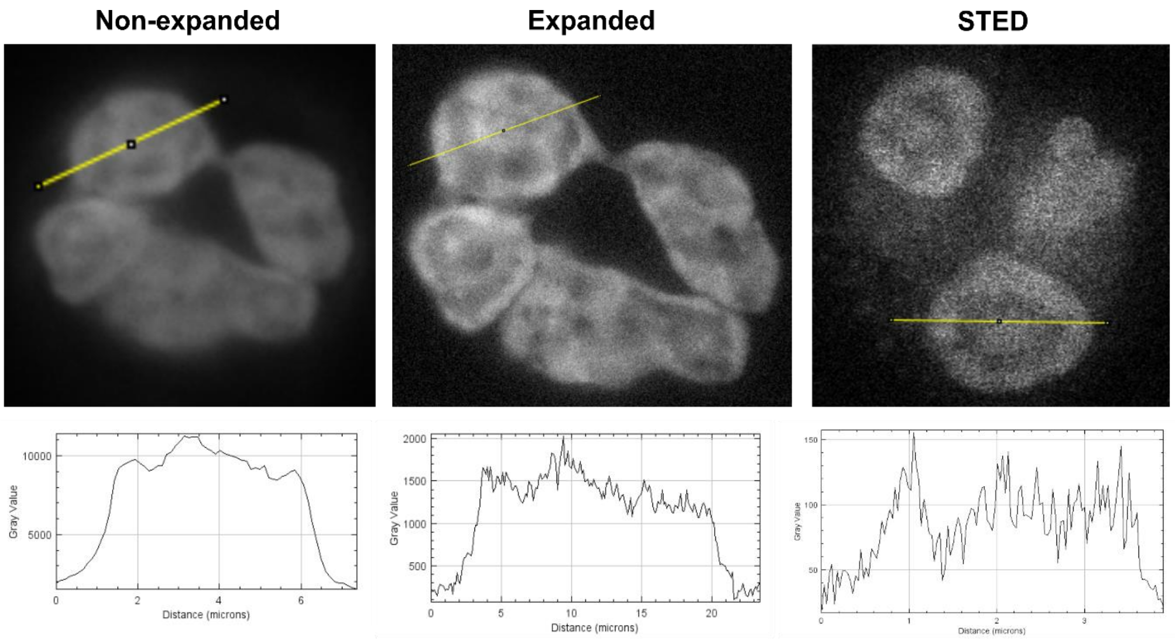

	Non-expanded	Expanded	STED
Percent maxima	11.59%	25.81%	31.30%

Supplementary Figure 10: Comparison of non-expanded, expanded, and STED percent maxima*. As a quantification of the level of detailed obtained in the above images, percent maxima was calculated by quantifying the number of peaks (individual local maximums corresponding to distinguishable points) and relating this to the total number of points measured by the respective line scan. There was a large gain in the level of detail with expansion, which was closer to the level observed with STED imaging. However, the level of detail obtained with STED is measured in typical (non-expanded) cells and so relatively this technique is still capable of a greater degree of super-resolution when strictly comparing to the gain in detail obtained with expansion.*

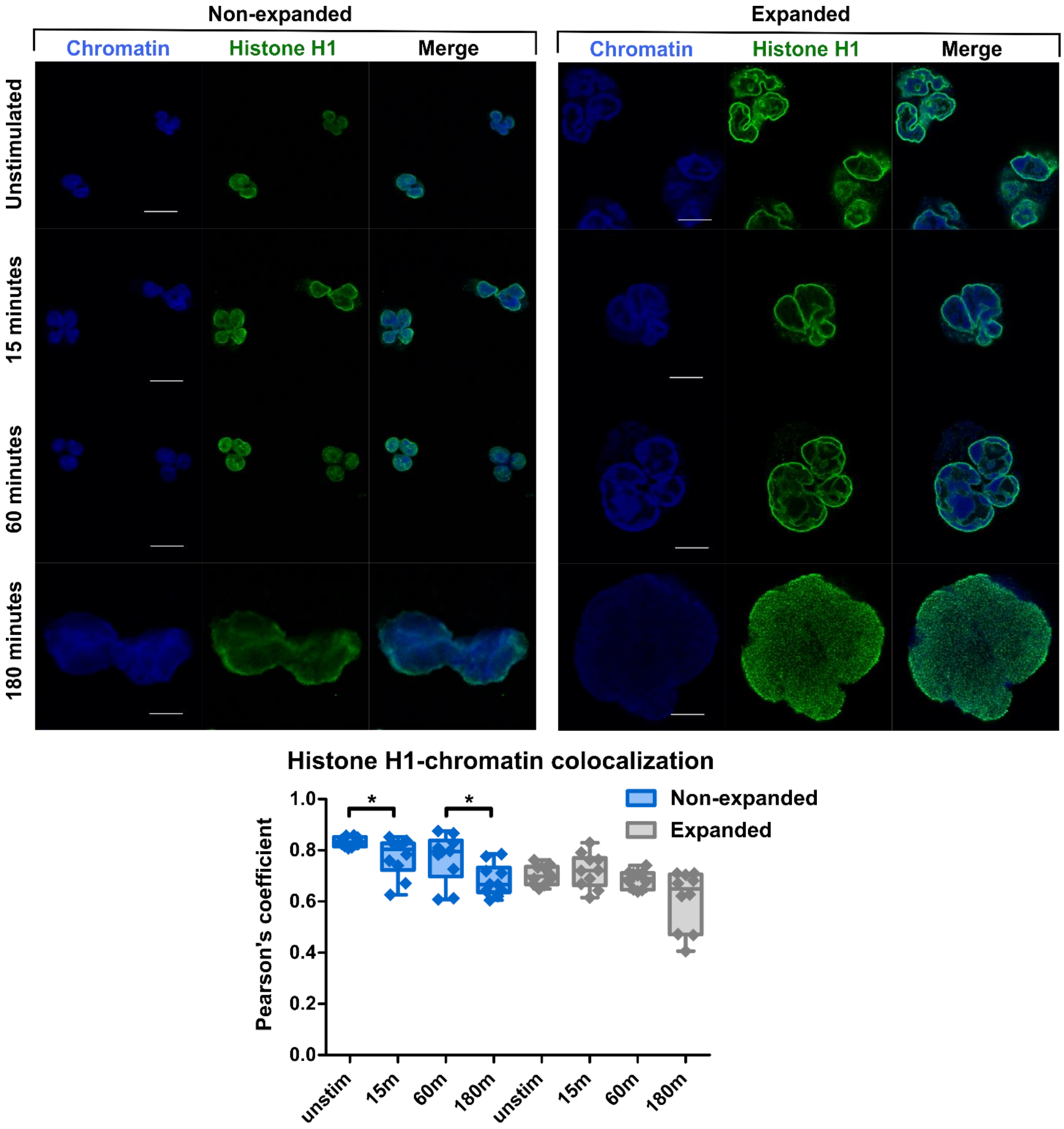

Supplementary Figure 11: Colocalization supplementary time series and measurements for histone H1 and chromatin*. Left shows non-expanded cells and right shows expanded cells. Graph is individual values (whiskers are minimum and maximum for each range); * = $p < 0.05$; n=10 cells analyzed per time point; images are PMA-stimulated human blood-derived neutrophils (100 nM), confocal images (63x); scale bars are 10 µm.*

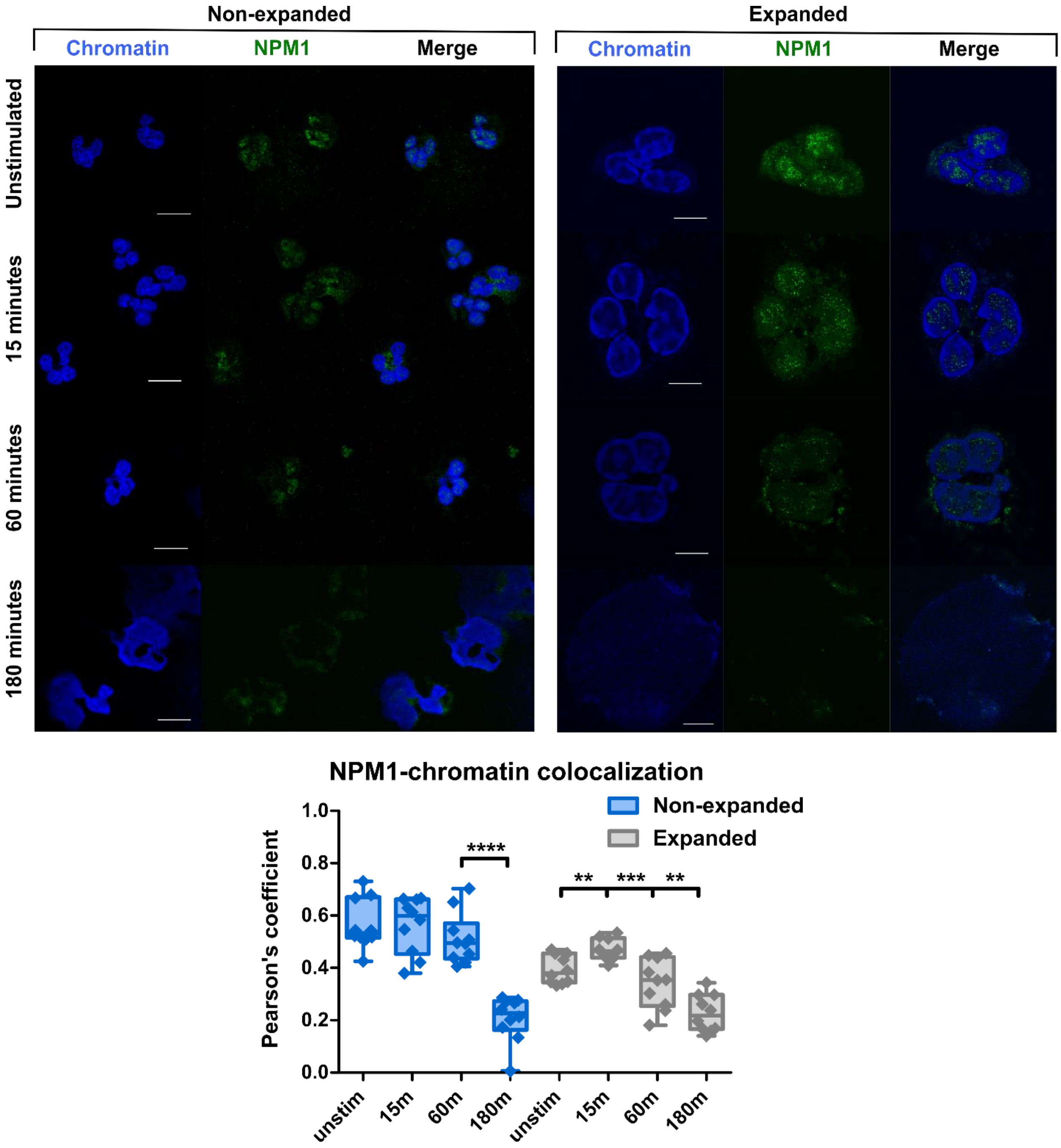

Supplementary Figure 12: Colocalization supplementary time series and measurements for NPM1 and chromatin*. Left shows non-expanded cells and right shows expanded cells. Graph is individual values (whiskers are minimum and maximum for each range); ** = p < 0.01, *** = p < 0.001, **** = p < 0.0001; n=10 cells analyzed per timepoint; images are PMA-stimulated human blood-derived neutrophils (100 nM), confocal images (63x); scale bars are 10 µm.*
