# Supplementary material for: Expansion microscopy of neutrophil nuclear structure and extracellular traps
Source: Biophys Rep (N Y). 2022 Dec 10;3(1):100091. doi: 10.1016/j.bpr.2022.100091 (PMC9813678; doi:10.1016/j.bpr.2022.100091)
Supplement: Document S2. Article plus supporting material [file mmc2.pdf]

Biophysical Reports

ARTICLE

Expansion microscopy of neutrophil nuclear structure and extracellular traps

Jason Scott Holsapple,[1] Lena Schnitzler,[2] Louisa Rusch,[3] Tobias Horst Baldeweg,[3] Elsa Neubert,[4] Sebastian Kruss,[2,5,6,*] and Luise Erpenbeck[1,*]

[1]Department of Dermatology, University Hospital Münster, Münster, Germany; [2]Department of Chemistry, Ruhr-University Bochum, Bochum, Germany; [3]Department of Dermatology, University Medical Center Göttingen, Göttingen, Germany; [4]Leiden Academic Centre for Drug Research, Leiden University, Leiden, the Netherlands; [5]Fraunhofer Institute for Microelectronic Circuits and Systems, Duisburg, Germany; and [6]Center for Nanointegration Duisburg-Essen (CENIDE), Duisburg, Germany

ABSTRACT Neutrophils are key players of the immune system and possess an arsenal of effector functions, including the ability to form and expel neutrophil extracellular traps (NETs) in a process termed NETosis. During NETosis, the nuclear DNA/chromatin expands until it fills the whole cell and is released into the extracellular space. NETs are composed of DNA decorated with histones, proteins, or peptides, and NETosis is implicated in many diseases. Resolving the structure of the nucleus in great detail is essential to understand the underlying processes, but so far, superresolution methods have not been applied. Here, we developed an expansion-microscopy-based method and determined the spatial distribution of chromatin/DNA, histone H1, and nucleophosmin with an over fourfold improved resolution (<40–50 nm) and increased information content. It allowed us to identify the punctate localization of nucleophosmin in the nucleus and histone-rich domains in NETotic cells with a size of 54–66 nm. The technique could also be applied to components of the nuclear envelope (lamins B1 and B2) and myeloperoxidase, providing a complete picture of nuclear composition and structure. In conclusion, expansion microscopy enables superresolved imaging of the highly dynamic structure of nuclei in immune cells.

WHY IT MATTERS Accessibility to high-resolution imaging is critical to advancing research across various disciplines. However, conventionally, this requires demanding optical hardware, special fluorophores, or data analysis. Expansion microscopy is a technique adaptable to different cell and tissue types and is comparatively inexpensive and easy to perform. Applying this technique to cells and compartments such as the nucleus of immune cells that are difficult to image due to their size and morphology yields valuable structural insights that would otherwise require more difficult superresolution methods.

INTRODUCTION

Neutrophilic granulocytes are an essential part of the innate immune system and comprise the most abundant type of granulocytes, making up 40%–70% of all white blood cells in humans. In many ways, neutrophils possess exceptional properties that allow them to migrate quickly to the place of inflammation, squeeze through the endothelium, and initiate immune responses (1–3).

Over the last years, it has become increasingly clear that the rate-limiting factor for cellular mobility is nuclear morphology and the biomechanics of nuclear deformation. Neutrophils possess an exceptionally deformable nucleus with a unique composition of the nuclear envelope. Mature neutrophils lack lamins A/C, which have been shown to be essential for mechanotransduction during confinement (4,5), and only contain low amounts of lamins B1 and B2 (6–8), which may constitute the mechanistic basis for the morphological plasticity of the neutrophil nucleus. Furthermore, the exceptional composition of the neutrophil nucleus also facilitates the formation of NETs (9–11), an immune-defense mechanism during which the entire neutrophil chromatin decondenses and expands first within the nuclear envelope and then, after rupture of said envelope, within the cytoplasm (12). Ultimately,

Submitted July 16, 2022, and accepted for publication December 7, 2022.

*Correspondence: sebastian.kruss@rub.de or luise.erpenbeck@ukmuenster.de

Jason Scott Holsapple and Lena Schnitzler contributed equally to this work.

Editor: Jorg Enderlein.

https://doi.org/10.1016/j.bpr.2022.100091

© 2022 The Authors.

This is an open access article under the CC BY license (http://creativecommons.org/licenses/by/4.0/).

the neutrophil chromatin, decorated with a multitude of antimicrobial peptides and enzymes, is expelled into the extracellular space, where it can immobilize and eliminate diverse pathogens such as bacteria, fungi, or even viruses (13). When neutrophil extracellular trap (NET) formation becomes dysregulated, however, it is implicated in several conditions including cancer metastasis, autoimmune diseases (14–16), and even severe COVID-19 (17). There are many factors that can modulate this process, including adhesion and substrate elasticity (9), ultraviolet light exposure (10), and the presence of serum proteins such as albumin (11). The physical properties of chromatin also importantly contribute to the formation and release of the NET due to the entropic swelling that occurs during the decondensation phase of NETosis (7,13,14,18).

Despite the apparent importance of the nuclear composition for its immune-defense functions and for the formation of NETs, studying nuclear functions and morphology remains difficult, as this requires very high-resolution imaging up to superresolution, which is technically challenging, time consuming, and costly. Of note, neutrophils are comparatively small cells (approximately 10 μm diameter) with an even smaller and anisotropic nucleus (2 μm). Thus, changes in the distribution of chromatin and nuclear proteins during NETosis are yet to be fully understood.

Superresolution methods have seen a tremendous progress in the past years and include stimulated emission depletion (STED) (19–21), photoactivated localization microscopy, stochastic optical reconstruction microscopy (22), superresolution optical fluctuation imaging (23), and Miniflux (24). These methods enable resolving biological structures far below the optical resolution limit but are complex in terms of the optical hardware or the necessary data processing (25–31). An alternative method is to increase the distance between fluorophores by expanding the sample (expansion microscopy). Expansion microscopy is a novel method wherein cells or tissues are embedded in a matrix and isotropically expanded (32–35). This process produces a nearly transparent sample with a gain in resolution that renders conventional microscopy a viable means to obtain superresolved images (36–39). This has been used to explore intracellular dynamics but also to further understand the interface between cells and their attachment matrix (40).

Here, we have developed a method to analyze neutrophils and their organelles such as the nucleus by expansion microscopy, achieving up to around fivefold higher resolution than the Abbe limit. The nucleus of neutrophils is especially interesting for such methods because, as outlined above, it is highly heterogeneous and responsive. We used this approach to study the distribution of histone H1 as well as lamins B1 and B2 within the nuclear envelope, nucleophosmin (NPM1), and to image characteristic neutrophilic molecules such as myeloperoxidase (MPO). Furthermore, we characterized neutrophil chromatin composition and histone distribution in unstimulated cells and in neutrophils undergoing NETosis. Along with a higher resolution, expansion microscopy of neutrophils yields a higher spatial separation of nuclear structures, revealing more details about their distribution and composition.

MATERIALS AND METHODS

Neutrophil isolation

All experiments with human neutrophils were approved by the Ethics Committee of the University Medical Center Göttingen (protocol number: 29/1/17). Neutrophils were isolated from fresh venous blood of healthy donors. Beforehand, all donors were fully informed about possible risks, and the informed consent was obtained in writing; consent could be withdrawn at any time during the study. Blood was received in S-Monovettes EDTA (7.5 mL, Sarstedt, Sarstedt, Germany) and neutrophils isolated according to previously published standard protocols (12,41). Neutrophils were resuspended in 1 mL HBSS$^{-Ca2+/Mg2+}$. Cells were counted and further diluted at the required concentration for the following procedures in RPMI 1640 containing 10 mM HEPES (Roth, Watertown, NY, USA) and 0.5% human serum albumin (Sigma-Aldrich, Burlington, MA, USA). Purity of the isolation was assessed by a cytospin assay (Cytospin 2 Zentrifuge, Shandon, Runcorn, UK) and Diff Quick staining (Medion Diagnostics, West Bengal, India). Cell purity was always greater than 98%.

NET induction

Fresh isolated human neutrophils were seeded in two eight-well, glass-bottom chamber slides with removable chamber (Thermo Fisher Nunc Lab-Tek II Chamber Slide System, Thermo Fisher Scientific, Waltham, MA, USA) at 80,000 cells/well in 200 μL RPMI 1640 (Lonza, Basel, Switzerland) containing 10 mM HEPES (Roth) and 0.5% FCS (Biochrom, Merck Millipore, Burlington, MA, USA). One chamber slide was used for gel expansion and the other one as a non-expanded control. The chamber slides were incubated for attachment for 30 min at 37°C. For NET formation, cells were activated with 100 nM phorbol 12-myristate 13-acetate (Sigma-Aldrich) for a defined period (15, 30, or 60 min, as indicated) while incubated at 37°C and 5% CO_2. To stop NETosis, cells were fixed with 3.2% paraformaldehyde and 0.1% glutaraldehyde in phosphate-buffered saline (PBS; Sigma-Aldrich) as final concentrations. The fixed samples were washed with PBS 200 μL/well. Then, cells were incubated in 100 mM glycine solution for 5 min at room temperature. Cells were washed again twice with PBS and stored overnight at 4°C.

Immunofluorescence staining

Before the staining procedure, the silicone sealing gasket was removed from the chamber slides. To block unspecific antibody binding, cells were incubated with blocking/permeabilization buffer (PBS with 0.1% Triton X-100 and 15% BSA) for 30 min at room temperature. Subsequently, cells were stained with polyclonal anti-human lamin B1 (immunoglobulin G [IgG], rabbit, 1:50) (ab16048, Abcam, Waltham, MA, USA); monoclonal anti-human lamin B2 (IgG2a, mouse, 1:100) (MA5-17274, Invitrogen, Waltham, MA, USA); polyclonal anti-human

MPO (IgG, sheep, 1:50) (AA16-718, Antibody Online, Pottstown, PA, USA); monoclonal anti-human histone H1 (IgG2a, mouse, 1:100) (MA5-13750, Invitrogen); monoclonal anti-human NPM1 (IgG1, mouse, 1:500) (325200, Invitrogen); or respective isotype controls (Fig. S1) in blocking/permeabilization buffer for 120 min, washed three times with PBS, and visualized with polyclonal anti-rabbit Alexa 488 (IgG, goat, 1:50) (ab11034, Abcam); polyclonal anti-mouse Alexa 488 (IgG, goat, 1:2,000) (A11029, Invitrogen); polyclonal anti-mouse Alexa 555 (IgG, goat, 1:50) (A21422, Life Technologies, Carlsbad, CA, USA); or anti-sheep Alexa 568 (IgG, donkey, 1:50) (ab175712, Abcam) as a secondary antibody in blocking/permeabilization buffer for 60 min at 37°C. After three more washes with PBS, chromatin was stained with Hoechst 33342 (1:2,000 in PBS) (Thermo Fisher) for 15 min at room temperature. The nonexpanded probes were mounted with Faramount Mounting Medium (Dako Agilent Technologies, Santa Clara, CA) on a coverslip. The probes for expansion were mounted with PBS and stored over night at 4°C.

Expansion microscopy

The protocol was modified from the previously published protocol from Chozinski et al. (42). Neutrophils were prepared as described above. The silicone sealing gasket of the chamber slide was removed from the microscope slide. Blocking and staining were carried out as described above. Stained cells were fixed with 0.25% glutaraldehyde in PBS for 10 min at room temperature. After washing in PBS, cells were incubated in monomer solution (1× PBS, 2 M NaCl, 2.5%/0.15% acrylamide/N'N'-methylenebisacrylamide, 8.625% sodium acrylate) for 1 min at room temperature. The gelation chamber was assembled by attaching three coverslips with water drops on a glass slide, forming a three-sided chamber (Fig. S2). Gelation solution was prepared by adding 10% TEMED (tetramethylethylenediamine) and 10% APS (ammonium persulfate) to the monomer solution to final concentrations of 0.2%. Next, a drop of 1,000 μL gelation solution was quickly put into the chamber. The chamber slide was laid slowly onto the drop with the cell side facing the drop and the edges resting on top of the chamber coverslips. The solution was allowed to gelate for 30 min at room temperature. Subsequently, the gel was removed together with the chamber slide from the gelation chamber and cut into eight pieces, corresponding to the former eight wells. Each gel was incubated in digestion buffer (8 U/mL proteinase K in 1× TAE [Tris base, acetic acid, EDTA], 10% Triton X-100, and 8% guanidine HCl) for 30 min at 37°C. The gels were then expanded in distilled water. The water was exchanged four times in 30 min intervals. After the last exchange, the gel was placed on a 35 mm imaging glass-bottom dish (Fig. S3). The samples were imaged with the Axiovert 200 or the Axio Imager M1 microscope or with a confocal laser scanning microscope (Olympus IX83 inverted microscope, software: Olympus Fluoview v.4.2; see below). Area and eccentricity of the nuclei were measured with ImageJ. Eccentricity was calculated as

$$\frac{4 \cdot area}{\pi \cdot majoraxis^2}.$$

Confocal microscopy

For confocal images, an Olympus IX83 inverted microscope (software: Olympus Fluoview v.4.2, Olympus, objective UPLFLN60XOI) was used. Hoechst fluorescence was excited at 405 nm; lamin B1, histone H1, and NPM1 fluorescence at 488 nm; and lamin B2 and MPO fluorescence at 568 nm. All pictures were further processed with ImageJ (v.1.53g, National Institutes of Health, Bethesda, MD, USA) and MATLAB (v.R2019a or v.R2020a, MathWorks, Natick, MA, USA).

Parameter calculations

All image processing for parameter calculations was conducted with Python. First, single cells from the microscope images were cropped, and then a threshold was applied to the DNA-stained images to create a mask that only included the staining intensities of the neutrophils and blocked all noise. These masks were applied to the corresponding DNA and histone images. These images could then be used for parameter calculation.

We also calculated the binning of the expanded microscope images by averaging 4 × 4 pixels to one to simulate the effect of the at least four times lower resolution of nonexpanded microscope images compared with expansion microscopy images. The Pearson coefficient was calculated with the Python SciPy library (scipy.stats).

Statistics

The colocalization of DNA and histone was calculated with the Pearson's coefficient. For all statistical significance tests, either the Mann-Whitney U test (using the scipy.stats package in Python, v.3.8.5, 64 bit) or unpaired *t*-test (using GraphPad Prism 5, v.5.04, 95% confidence intervals) was performed. The letter "n" indicates the number of independent experiments from individual donors. For every donor and all conditions, at least 40 cells were evaluated in a blinded manner.

RESULTS

Expansion microscopy of primary human neutrophils and NETs

Building upon previous protocols for expansion microscopy of cultured cells and histological samples (43,44), we developed a technique for the staining and visualization of single primary human immune cells and, specifically, for neutrophilic granulocytes.

Freshly isolated human neutrophils were placed in chamber slides and activated to form NETs. They were then fixed and stained by using standard immunofluorescence techniques. Following a second fixation step with glutaraldehyde, cells were then engulfed in monomer solution, and the gelation process was initiated (Fig. 1 *A*) on top of a glass coverslip. The resulting gel samples were incubated in digestion buffer containing proteinases and finally placed in distilled water to initiate the swelling process. After overnight expansion of the gel, cells within the sample were ready to be visualized either by conventional wide-field or confocal microscopy (Fig. 1, *B* and *C*). Alternatively, by using eight-well chamber slides for the initial settling of the neutrophils and then later cutting the gel into eight pieces according to the previous borders of the chambers, it was also possible to perform up to eight different stainings of the cells in the respective chambers (Fig. S4).

After establishing the general feasibility of this protocol, we assessed the magnification we were able to reach using freshly isolated neutrophils that were stained with the nuclear dye Hoechst. In one dimension, a 4.9-fold magnification was achieved for the

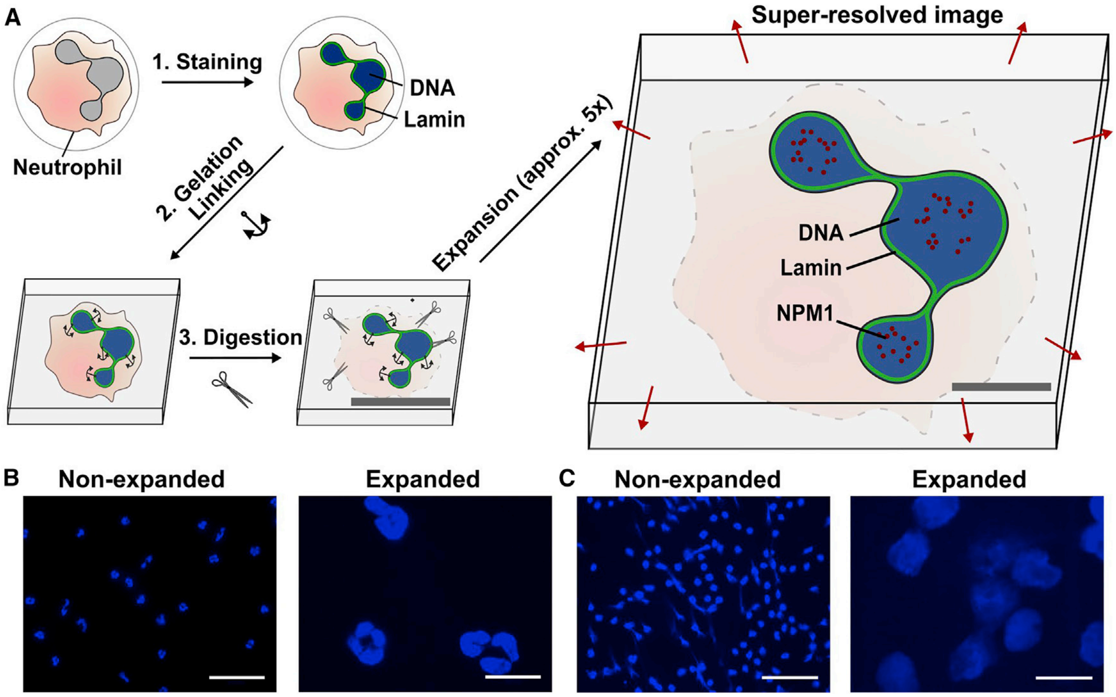

FIGURE 1 Expansion microscopy of neutrophils and NETs. Schematic of the method (*A*): cells are stained with fluorescently labeled antibodies or common fluorescence dyes (e.g., Hoechst). They are then embedded in a polyacrylamide gel, and proteins are digested. Afterward, the gel is isotropically expanded via swelling in distilled water. Representative confocal microscopy images of Hoechst-stained nonexpanded or expanded neutrophils (*B*) and NETs (*C*). Scale bars are 10 (*A*), 50 (*B*), and 100 μm (*C*).

stained nuclei, amounting to a 25-fold increase in chromatin-stained area in the 2D images (Fig. 2 *A*). To study cellular morphology with this novel method, it is important that the proportions are maintained during mechanical enlargement of the previously stained structures. For this reason, we also assessed whether expansion of the nuclei was isotropic, and thus that it is uniform in all directions. To this end, we determined eccentricity of the stained nuclei before and after expansion and found that eccentricity did not vary between expanded and nonexpanded nuclei (Fig. 2 *B*). We thus confirmed that the proportions of stained structures were not altered by expansion microscopy.

In addition to neutrophil nuclei, we also stained NETs by Hoechst and observed them after following the protocol of expansion microscopy. As NETs are composed of strands of decondensed chromatin decorated with a variety of peptides and proteins, they are very fragile and often subject to mechanical artifacts during staining procedures. Here, we could show that NETs could be visualized similarly well as nonexpanded nuclei using the above-explained staining, fixation, and expansion procedure. A 4.5-fold magnification was again achieved by expansion (Fig. 2 *A*), while eccentricity of NETs did not change after the procedure (Fig. 2 *B*). Thus, expansion microscopy is suited even for the visualization of fragile cellular structures.

To further validate the isotropy of expansion, it was necessary to both measure the expansion factor in the same cells pre- and postexpansion and assess if the distribution of chromatin was affected by the expansion process. To address the first point, 10 cells were selected, and their nuclear areas measured pre- and postexpansion (Fig. 2 *C*). The expansion factor was again determined by dividing the postexpansion area by the preexpansion area, and the values for the 10 cells were averaged to obtain an expansion factor of 4.06 overall. This is within the range observed in the literature for this technique and for gel composition. The slight difference in expansion factor between different (Fig. 2 *A*) and the same (Fig. 2 *C*) cells might reflect the overall variation of the expansion process and cell size variations between different cells. This again reflects the heterogeneity of neutrophil nuclei.

To address chromatin distribution pre- and postexpansion, we performed dimensional analyses using line scans of the same cell before and after the expansion process (Fig. S5). Cumulative plots were produced to assess if there was any change in the relative distribution of chromatin due to the procedure, and the highly similar slopes of the regression lines suggest that this is largely unaffected. Additionally, correlation analyses were performed to compare nonexpanded and expanded line scans. Cross-correlation analysis

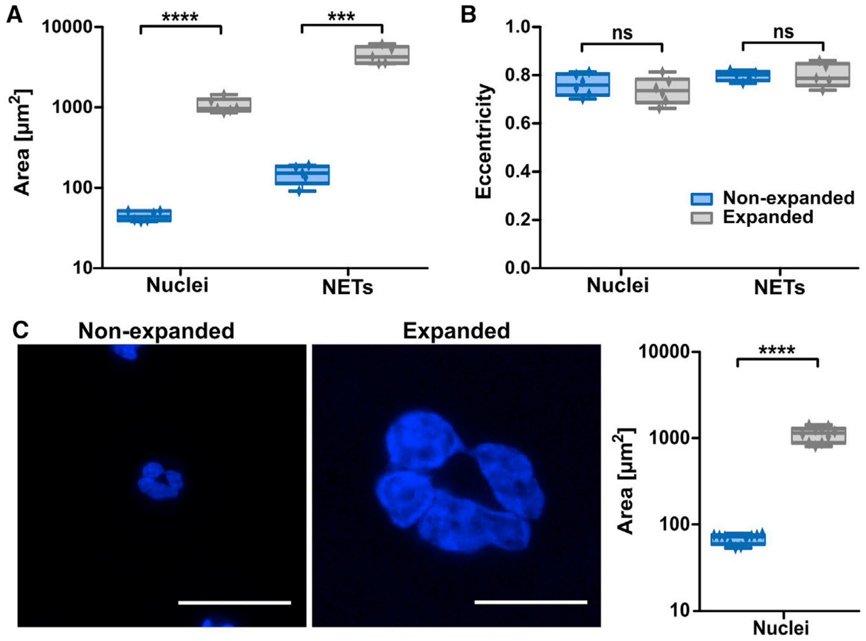

FIGURE 2 Isotropy of expansion of neutrophil nuclei and NETs. With expansion, the areas of different neutrophil nuclei (n = 6) or NETs (n = 5) increased (*A*) in contrast to eccentricity of nuclei or NETs, which was constant (*B*). The mean nuclear area increased from 44.8 to 1,060 μm^2, amounting to a lateral expansion of 4.9. When the same cells (n = 10) were identified and measured pre- and postexpansion (*C*), the mean nuclear area increased from 68 to 1,119 μm^2, amounting to a lateral expansion of 4.06. Scale bars are 25 μm. Boxplot shows interquartile range, mean values, and minimum to maximum (*whiskers*). Significance was tested using unpaired *t*-tests, ***$p \leq 0.001$, ****$p \leq 0.0001$.

produced clear peaks, suggesting a high degree of correlation between the line scans (Figs. S5 and S6). Together, these measurements suggest that the expansion procedure does not significantly alter the distribution of chromatin.

Expansion microscopy significantly improves resolution and provides more information on spatial distribution of nuclear structures

Next, we assessed whether expansion microscopy provided measurable improvements over nonexpanded cells. To this end, we analyzed line scans through representative images of unstimulated neutrophils that had been stained for DNA, histone H1, and NPM1 and subsequently processed in Python (Fig. 3). The line scan through neutrophil nuclear lobules revealed intensity signals for all three stainings throughout the cell. In unstimulated neutrophil nuclei, chromatin staining was moderately enhanced toward the periphery of the nucleus, while histone H1 localized strongly towards the periphery of the nucleus.

In contrast, NPM1 was distributed throughout the lobules of the nucleus. As a theoretical construct to assess the advantages of expansion microscopy, we calculated binned images of the expanded microscope images by averaging an area of 4 × 4 pixels to one to simulate the effect of the at least four times lower resolution of nonexpanded microscope images compared with expansion microscopy images.

In the line scans (Fig. 3), an increase of resolution by expansion microscopy images compared with normal, nonexpanded microscopy and with the binned cells is clearly visible by the higher density of information and the increase in details on intensity distribution. It is important to note that the expansion factor cannot be determined by comparing the length of the line scans of different cells due to the heterogeneity of the individual lobes. This would only be applicable in comparing the same fixed cell pre- and postexpansion.

In a next step, colocalization of DNA and histones was then calculated using the Pearson's coefficient from the statistical functions of the Python SciPy library (Fig. 4). Colocalization was assessed in unstimulated expanded, nonexpanded, and "binned" cells (see above). A lower coefficient indicates a higher degree of spatial separation between stained structures. Of note, in the expanded images, a significantly lower Pearson's coefficient was shown for expanded versus nonexpanded cells, reflecting the above-described enhancement of histone H1 at the border of the chromatin-stained area (as shown in Figs. 3 *A*, 5, and S7). In conclusion, nonexpanded images provided much less information about distinct localization of DNA and histones, as can be derived from the Pearson's coefficient that was close to 1. For the binned images, Pearson's coefficient was in the same range as for the nonexpanded cells, as was to be expected because information regarding colocalization is lost in the binning process. Since the Pearson's coefficient of binned and nonexpanded images are in the same range, the artificial loss induced with the binning seems also to be similar to the fourfold difference in resolution between expanded and nonexpanded images.

Expansion dilutes the density of fluorophores, which could decrease the contrast of the image. In the case

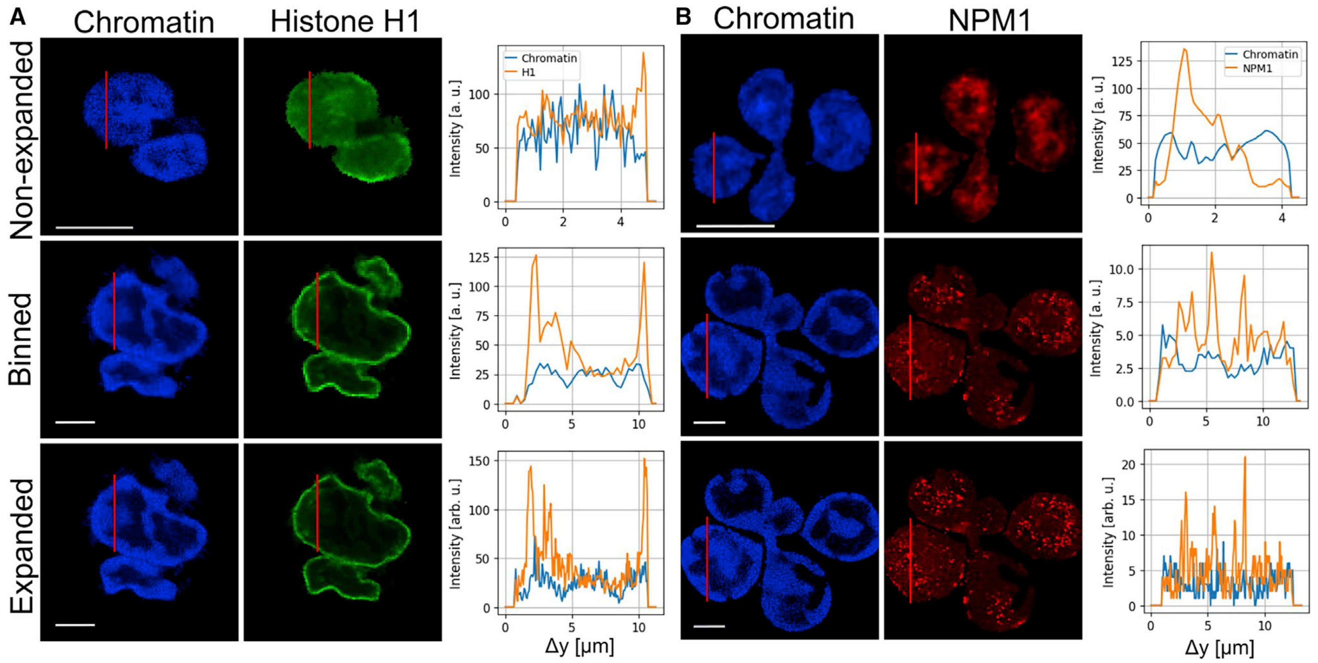

FIGURE 3 Distribution of chromatin and nuclear features in expanded and nonexpanded neutrophils. (*A*) Fluorescence images of Hoechst- (*left*) and histone H1-labeled (*middle*) neutrophils without expansion microscopy, binned (4 × 4) expansion microscopy images, and with expansion microscopy. The red line indicates a line scan through the nucleus and is shown on the right. Note that the cells shown for nonexpanded are not the same as for expanded/binned. All images show unstimulated cells. (*B*) Fluorescence images of Hoechst- (*left*) and NPM1-labeled (*middle*) neutrophils without expansion (*top*) microscopy, binned (4 × 4) expansion microscopy images (*middle*), and with expansion microscopy (*bottom*). The results indicate that there is more spatial information contained in the higher-resolution expansion microscopy images. All scale bars are 5 μm for expanded and nonexpanded.

of the Hoechst dye, the mean fluorescence intensity (at the same imaging conditions) decreased (Fig. 4 *B*) in proportion to the expansion factor. In contrast, the antibody-based histone H1 decreased, but to a smaller amount (Fig. S8), suggesting a certain level of proximity-based quenching of the fluorophores. In the case of NPM1, the fluorescence signal of the expanded sample increases. An explanation for this interesting observation could be that the loss of proximity-based quenching here outcompetes dilution because of expansion. This result could be explained by the punctate high-density localization of NPM1. However, multiple factors (for example, proximity and conformation of labeled structures as well as the physical properties of the fluorophore or the local chemical environment [pH] or modification of the fluorophores by the enzymes, etc., used in the expansion protocol) are involved in potential signal quenching and subsequent increase of fluorescence signal, which would be worth exploring in greater detail in the future.

Increase of spatial information in biological processes like NETosis

Next, we studied NETosis by conventional microscopy and by expansion microscopy after staining for DNA (Hoechst) and histone H1 (Fig. 5). The images revealed a preferential distribution of histone H1 toward the periphery of the nucleus, particularly at the interface between chromatin and the cytoplasm. Chromatin was similarly enriched toward the periphery of the nucleus, although less prominently compared with H1. During the process of NETosis, chromatin starts to expand within the cell (45). Typically, this also entails mixing of nuclear and cytoplasmic proteins with the chromatin. Interestingly, the histone H1 staining in expanded cells revealed the emergence of additional "interfaces" with an enhancement of H1 staining at 60 min (Fig. 5). In nonexpanded images, this was not visible, illustrating the gain of biological information by expansion microscopy.

In cells that are completely filled with DNA just before membrane rupture (Fig. 5, *first row on the right*), histones and DNA showed a rather even distribution of chromatin and histone H1. This agrees with previously performed STED microscopy that showed no fine structure of the chromatin (12). However, the higher resolution achieved with expansion enabled us to reveal a certain granularity of the histone H1 and DNA. These domains had a diameter 266 ± 45 nm (expanded), which corresponds to 54–66 nm before expansion (Fig. S9). This suggests that the DNA

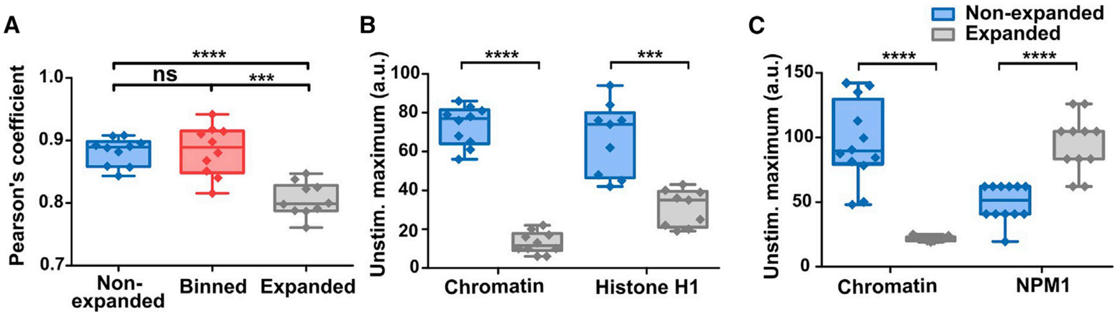

FIGURE 4 Impact of expansion on information content and maximum signal intensity. (*A*) The Pearson's coefficient serves as a measure for colocalization for unstimulated cells stained with Hoechst and H1 for expanded, binned (4 pixels), and nonexpanded images. Colocalization is smaller for expanded images indicating more information content. Statistical analysis was performed with the Mann-Whitney U test. ***p = 0.001, ****p = 0.0001, n.s. = not significant (n_expanded ≥ 10, n_nonexpanded ≥ 10). (*B* and *C*) Maximum signal intensity decreases with expansion in unstimulated cells stained for chromatin and histone H1 (*B*), while these values are relatively lower overall for NPM1. (*C*) Statistics were performed with the Mann-Whitney U test. ***p = 0.001, ****p = 0.0001, n.s. = not significant (n_expanded ≥ 9, n_nonexpanded ≥ 9). Boxplot shows interquartile range, mean values, and minimum to maximum (*whiskers*).

meshwork is not homogenous but rather like a soft material with more rigid spheres incorporated ("raisins in a cake"), and this should have implications for the mechanical properties of the cells.

Expansion microscopy is suited for the visualization of diverse cytoplasmic and nuclear neutrophil structure

To further assess the suitability of expansion microscopy of neutrophils for different cellular structures, we performed immunofluorescence stainings for cytoplasmic proteins (MPO) and components of the nuclear lamina (lamin B1 and B2). The staining of histone H1 and DNA had already been performed for Figs. 3 and 5. We were able to perform all immunofluorescence stainings and increase the resolution for both cytoplasmic as well as nuclear markers as seen in Figs. 6 and 7.

Thus, expansion microscopy is well suited for staining a large range of epitopes inside neutrophils and can serve as general tool to study these cells. Similar to the case for histone H1 (Fig. 5), the increased amount of spatial information allowed us to learn more about the localization of certain proteins. NPM1 shows punctate agglomerations inside the nucleus that were not visible in the nonexpanded cells (Fig. 7). In the example images below, these were measured to have areas of $0.09 \pm 0.03\ \mu m^2$ (at least 25 randomly selected particles after thresholding, mean ± SD). They could be attributed to NPM1-related liquid-liquid phase separation and could play a role for gene regulation.

DISCUSSION

Neutrophils are highly complex cells of the innate immune system. While originally considered a homogeneous population with a highly conserved repertoire of immune-defense mechanisms, recent research has been shedding light on neutrophil heterogeneity and functional versatility. Indeed, neutrophils have emerged as key players not only during acute and chronic inflammation but also in malignant diseases and have thus been propelled into the focus of inflammation and cancer research. Of note, the neutrophil nucleus provides neutrophils with unique properties and abilities, including the comparatively high propensity for migration.

The rekindled interest in neutrophil biology has revealed the need for efficient methods to characterize neutrophil composition at high resolution. In principle, this can be accomplished with superresolution techniques that are costly, time consuming, and require specialized equipment. For this reason, we developed a novel, low-cost, and generally accessible approach to superresolution fluorescence images of neutrophils. Instead of relying on optical techniques to circumvent the limit of resolution, expansion microscopy enlarges the cellular structures by embedding the cells in a polyacrylamide hydrogel and then letting the hydrogel swell. Importantly, we have shown that the swelling process enlarges structures in a homogeneous manner without altering eccentricity (Fig. 2). This is especially important in light of the lobulated morphology of nuclei in neutrophils. While the area is increased, the spatial relationship between structures is maintained. This was not only true for intact neutrophils but also for NETs, highlighting the general suitability of this method even for fragile structures.

To quantitate the improvement in resolution gained by expansion microscopy, we stained chromatin/DNA by a Hoechst dye and histone H1 or NPM1 by a fluorescently labeled antibody. As it was not possible to directly compare the same cell in an expanded versus a nonexpanded state, we simulated a 4.9-fold decreased resolution in images of expanded cells by binning of 4 pixels (Fig. 3, *binned*) to provide a direct

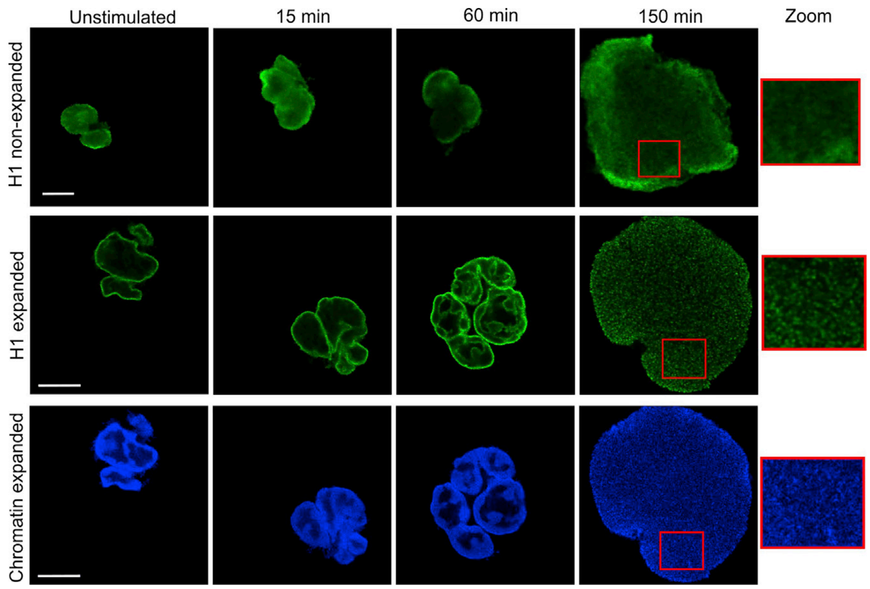

FIGURE 5 Identification of histone-rich domains below the resolution limit. Exemplary images of neutrophils undergoing NET formation after activation with 100 nM PMA. DNA was stained by Hoechst (*bottom row*) and histone H1 (*top and middle rows*). In the first row, nonexpanded cells are shown as a comparison with the expanded cells in the second and third rows. DNA and histone H1 colocalize during the first part of NET formation. For NETotic cells before membrane rupture (150 min), an enlarged region of interest is shown, illustrating granularity and histone-rich domains with higher DNA density. Scale bars are 10 μm for expanded and 5 μm for nonexpanded images.

measure for the improvement of resolution. Of note, this measure is similar to the gain in resolution.

We were able to show that the density of information across the neutrophil nucleus strongly increased by expansion microscopy (Figs. 3, *A* and *B*, and S10), as depicted in line scans along the axis of the nucleus and comparison with STED imaging of neutrophil nuclei. In the expanded cell, the line scan had shown a much higher amplitude and frequency of excursions as a measure for the gain of structural information. Expansion improved the (theoretical) resolution from 163 to 34 nm (Hoechst dye).

Additionally, we calculated optical colocalization in nonexpanded cells and expanded cells. In the nonexpanded cells, differences in distribution cannot be assessed due to the limit in resolution, resulting in a Pearson's coefficient closer to 1. Expansion microscopy significantly lowered the Pearson's coefficient, as shown by Mann-Whitney U tests in Fig. 4. Although chromatin and histone H1 or NPM1 are located together in the nucleus, their distribution is not identical (Figs. 3, *A* and *B*, 7, S11, and S12), which is in line with previously published literature (46,47). These differences were suitably imaged by expansion microscopy, where conventional microscopy with nonexpanded cells failed to show differences.

As a further proof of suitability of this method for neutrophils, we stained different neutrophil structures such as the nuclear lamins B1 and B2 and the cytoplasmic protein MPO (Fig. 6), in addition to histone H1 and NPM1 (Figs. 3, 5, and 7). All structures were imageable by the same expansion microscopy protocol. Strikingly, NPM1 appears as punctate "mininucleoli" upon expansion, which has been discussed in the literature but not revealed in this detail (48). As shown in Fig. S12, NPM1 had a lower degree of colocalization with chromatin upon expansion. This, together with the line scan analysis in Fig. 3, showed the distribution of NPM1 as being more prevalent in the less-dense chromatin regions. Expanded histone H1 also strongly appeared in clearly defined border zones, often described as lamina-associated domains between heterochromatin and the nuclear lamina (49). This is reflected in Fig. S11, as the colocalization measurements between histone H1 and chromatin decreased upon expansion, showing both the peripheral localization and granularity of this nuclear protein. However, it should be noted that in the case of lamin B2, staining was somewhat blurred around the nucleus, especially in contrast to the very clear, yet weaker, lamin B1 staining. It is conceivable that the expansion process also led to a widening of the nuclear lamina and/or a certain washing out of the fluorophores. In the case of lamin B1, we observed the need for relatively high amounts of antibodies to achieve a good signal. As the expansion process does not increase epitopes but, conversely, "dilutes" them within the hydrogel, fluorophores with a good intensity are of high importance for this method. This is also reflected in the intensity of the staining in the line scans of Fig. 3, as well as the lower signal intensities for most labels. Thus, expansion microscopy comes with the price of losing staining intensity while gaining resolution. In any case, the staining procedure should be adjusted for any new antibody/fluorophore that is to be used in combination with expansion microscopy, especially if one aims to study proteins that are not expressed in large quantities in the cell.

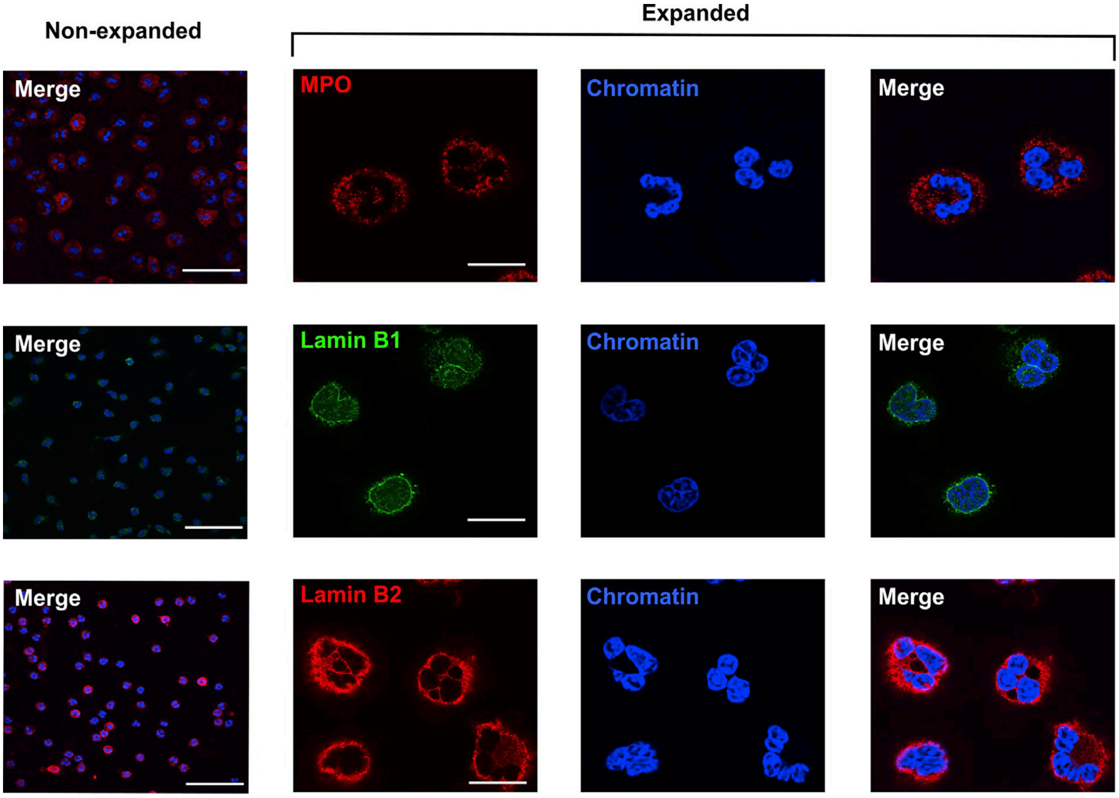

FIGURE 6 Expansion microscopy and immunofluorescence staining of neutrophil cytoplasmic and nuclear lamina proteins. MPO (first row) was stained as a characteristic cytoplasmic protein, and lamin B1 (*second row*) and lamin B2 (*third row*) as components of the nuclear lamina of neutrophils. Column 1 shows nonexpanded cells. Columns 2–4 show expanded cells, labeled as indicated. Scale bars are 50 μm.

While we have used pregelation labeling in our current study, postgelation labeling is an alternative method wherein the target structures are exposed to antibodies following the gelation and digestion steps. Utilizing pre- or postgelation labeling is largely dependent on the stability of the fluorophore versus the epitope in terms of the digestion and expansion. If a fluorophore is known to be highly susceptible to degradation by proteinase K, then postgelation labeling would be the optimal choice; however, our method of pregelation labeling uses significantly less antibody, takes less staining time, and is suitable for epitopes that are likely to degraded by digestion. Additionally, we primarily used fluorophores of the GFP family, which are resistant to degradation from this type of digestion.

CONCLUSION

In summary, we have demonstrated expansion microscopy for human neutrophils for different epitopes

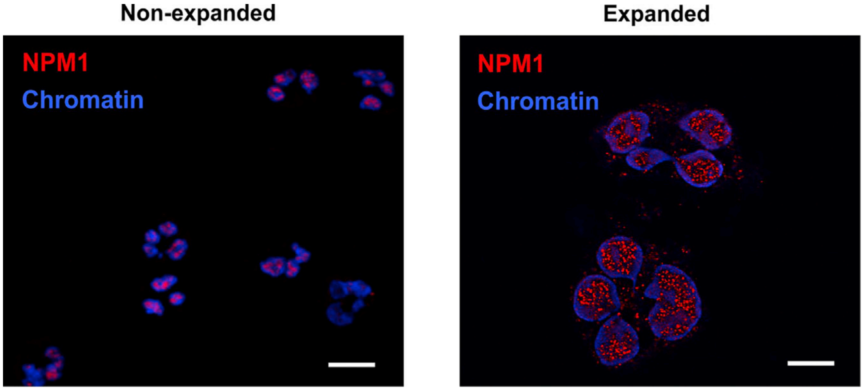

FIGURE 7 Expansion microscopy reveals punctate protein agglomerations in the neutrophil nucleus. NPM1 was stained as a nuclear protein involved in unique higher-order structural conformations. Left shows nonexpanded cells and right shows expanded cells. The enhancement in resolution shows that NPM1 is not evenly distributed and appears as puncta within the chromatin of the nucleus. Scale bars are 10 μm.

and fluorescent labeling strategies, including dyes (Hoechst) and antibody-based labeling. The increase in resolution allowed us to achieve a deeper understanding of the nuclear morphology and the nanoscale topography of chromatin and nuclear proteins. Therefore, expansion microscopy allows to better understand the complex and difficult-to-image structure of nuclei of highly dynamic cells such as human neutrophils.

SUPPORTING MATERIAL

Supporting material can be found online at https://doi.org/10.1016/j.bpr.2022.100091.

AUTHOR CONTRIBUTIONS

S.K. and L.E. designed the project; J.S.H., L.R., and T.H.B. performed research; J.S.H., L.S., L.R., and T.H.B. analyzed data; J.S.H., L.S., L.R., and E.N. generated figures; J.S.H., L.S., L.R., E.N., S.K., and L.E. wrote the manuscript.

ACKNOWLEDGMENTS

This work was funded by the Deutsche Forschungsgemeinschaft (DFG; German Research Foundation) under Germany's Excellence Strategy-EXC 2033-390677874-RESOLV. We acknowledge support by the DFG via the Heisenberg program (S.K.) and a single grant. L.E. received support from the DFG (ER 321/2-1) and TRR332. J.S.H. is a member of the CiM-IMPRS graduate program Münster (CiM). This work is supported by the "Center for Solvation Science ZEMOS" funded by the German Federal Ministry of Education and Research BMBF and by the Ministry of Culture and Research of Nord Rhine-Westphalia. We thank Meike Steinert for technical support.

DECLARATION OF INTERESTS

The authors declare no competing interests.

REFERENCES

1. Ley, K., H. M. Hoffman, ..., S. D. Catz. 2018. Neutrophils: New insights and open questions. *Sci. Immunol.* 3:eaat4579. https://doi.org/10.1126/sciimmunol.aat4579.
2. Renkawitz, J., and M. Sixt. 2016. A Radical Break: Restraining Neutrophil Migration. *Dev. Cell.* 38:448–450. https://doi.org/10.1016/j.devcel.2016.08.017.
3. Gaertner, F., Z. Ahmad, ..., S. Massberg. 2017. Migrating Platelets Are Mechano-scavengers that Collect and Bundle Bacteria. *Cell.* 171:1368–1382.e23. https://doi.org/10.1016/j.cell.2017.11.001.
4. Lomakin, A. J., C. J. Cattin, ..., M. Piel. 2020. The nucleus acts as a ruler tailoring cell responses to spatial constraints. *Science.* 370:2894. https://doi.org/10.1126/science.aba2894.
5. Sorce, B., C. Escobedo, ..., D. J. Müller. 2015. Mitotic cells contract actomyosin cortex and generate pressure to round against or escape epithelial confinement. *Nat. Commun.* 6:8872. https://doi.org/10.1038/ncomms9872.
6. Olins, A. L., T. v. Hoang, ..., D. E. Olins. 2009. The LINC-less granulocyte nucleus. *Eur. J. Cell Biol.* 88:203–214. https://doi.org/10.1016/j.ejcb.2008.10.001.
7. Olins, A. L., M. Zwerger, ..., D. E. Olins. 2008. The human granulocyte nucleus: Unusual nuclear envelope and heterochromatin composition. *Eur. J. Cell Biol.* 87:279–290. https://doi.org/10.1016/j.ejcb.2008.02.007.
8. Rowat, A. C., D. E. Jaalouk, ..., J. Lammerding. 2013. Nuclear Envelope Composition Determines the Ability of Neutrophil-type Cells to Passage through Micron-scale Constrictions. *J. Biol. Chem.* 288:8610–8618. https://doi.org/10.1074/jbc.M112.441535.
9. Erpenbeck, L., A. L. Gruhn, ..., S. Kruss. 2019. Effect of Adhesion and Substrate Elasticity on Neutrophil Extracellular Trap Formation. *Front. Immunol.* 10:2320. https://doi.org/10.3389/fimmu.2019.02320.
10. Neubert, E., K. M. Bach, ..., L. Erpenbeck. 2019. Blue and Long-Wave Ultraviolet Light Induce in vitro Neutrophil Extracellular Trap (NET) Formation. *Front. Immunol.* 10:2428. https://doi.org/10.3389/fimmu.2019.02428.
11. Neubert, E., S. N. Senger-Sander, ..., L. Erpenbeck. 2019. Serum and Serum Albumin Inhibit in vitro Formation of Neutrophil Extracellular Traps (NETs). *Front. Immunol.* 10:12. https://doi.org/10.3389/fimmu.2019.00012.
12. Neubert, E., D. Meyer, ..., S. Kruss. 2018. Chromatin swelling drives neutrophil extracellular trap release. *Nat. Commun.* 9:3767. https://doi.org/10.1038/s41467-018-06263-5.
13. Brinkmann, V., U. Reichard, ..., A. Zychlinsky. 2004. Neutrophil extracellular traps kill bacteria. *Science.* 303:1532–1535. https://doi.org/10.1126/science.1092385.
14. Lin, A. M., C. J. Rubin, ..., A. T. Bruce. 2011. Mast cells and neutrophils release IL-17 through extracellular trap formation in psoriasis. *J. Immunol.* 187:490–500. https://doi.org/10.4049/jimmunol.1100123.
15. Knight, J. S., C. Carmona-Rivera, and M. J. Kaplan. 2012. Proteins derived from neutrophil extracellular traps may serve as self-antigens and mediate organ damage in autoimmune diseases. *Front. Immunol.* 3:380. https://doi.org/10.3389/fimmu.2012.00380.
16. Sur Chowdhury, C., S. Giaglis, ..., P. Hasler. 2014. Enhanced neutrophil extracellular trap generation in rheumatoid arthritis: analysis of underlying signal transduction pathways and potential diagnostic utility. *Arthritis Res. Ther.* 16:R122. https://doi.org/10.1186/ar4579.
17. Schön, M. P., C. Berking, ..., K. Steinbrink. 2020. COVID-19 and immunological regulations – from basic and translational aspects to clinical implications. *JDDG J. der Deutschen Dermatol. Gesellschaft.* 18:795–807. https://doi.org/10.1111/ddg.14169.
18. Thiam, H. R., S. L. Wong, ..., C. M. Waterman. 2020. NETosis proceeds by cytoskeleton and endomembrane disassembly and PAD4-mediated chromatin decondensation and nuclear envelope rupture. *Proc. Natl. Acad. Sci. USA.* 117:7326–7337. https://doi.org/10.1073/pnas.1909546117.
19. Eggeling, C., K. I. Willig, ..., S. W. Hell. 2015. Lens-based fluorescence nanoscopy. *Q. Rev. Biophys.* 48:178–243. https://doi.org/10.1017/S0033583514000146.
20. Göttfert, F., T. Pleiner, ..., S. W. Hell. 2017. Strong signal increase in STED fluorescence microscopy by imaging regions of subdiffraction extent. *Proc. Natl. Acad. Sci. USA.* 114:2125–2130. https://doi.org/10.1073/pnas.1621495114.
21. Kasper, R., B. Harke, ..., M. Sauer. 2010. Single-molecule STED microscopy with photostable organic fluorophores. *Small.* 6:1379–1384. https://doi.org/10.1002/smll.201000203.
22. Zwettler, F. U., S. Reinhard, and M. Sauer. 2021. Ex-dSTORM and automated quantitative image analysis of expanded filamentous structures. *Methods Cell Biol.* 161:317–340. https://doi.org/10.1016/bs.mcb.2020.05.004.

23. Dertinger, T., R. Colyer, …, J. Enderlein. 2009. Fast, background-free, 3D super-resolution optical fluctuation imaging (SOFI). *Proc. Natl. Acad. Sci. USA*. 106:22287–22292. https://doi.org/10.1073/pnas.0907866106.
24. Gwosch, K. C., J. K. Pape, …, S. W. Hell. 2020. MINFLUX nanoscopy delivers 3D multicolor nanometer resolution in cells. *Nat. Methods*. 17:217–224. https://doi.org/10.1038/s41592-019-0688-0.
25. Dankovich, T. M., and S. O. Rizzoli. 2021. Challenges facing quantitative large-scale optical super-resolution, and some simple solutions. *iScience*. 24:102134. https://doi.org/10.1016/j.isci.2021.102134.
26. Sednev, M. v., V. N. Belov, and S. W. Hell. 2015. Fluorescent dyes with large Stokes shifts for super-resolution optical microscopy of biological objects: a review. *Methods Appl. Fluoresc.* 3:042004. https://doi.org/10.1088/2050-6120/3/4/042004.
27. Sahl, S. J., S. W. Hell, and S. Jakobs. 2017. Fluorescence nanoscopy in cell biology. *Nat. Rev. Mol. Cell Biol.* 18:685–701. https://doi.org/10.1038/nrm.2017.71.
28. Sauer, M., and M. Heilemann. 2017. Single-Molecule Localization Microscopy in Eukaryotes. *Chem. Rev.* 117:7478–7509. https://doi.org/10.1021/acs.chemrev.6b00667.
29. Schermelleh, L., A. Ferrand, …, G. P. C. Drummen. 2019. Super-resolution microscopy demystified. *Nat. Cell Biol.* 21:72–84. https://doi.org/10.1038/s41556-018-0251-8.
30. Werner, C., M. Sauer, and C. Geis. 2021. Super-resolving Microscopy in Neuroscience. *Chem. Rev.* 121:11971–12015. https://doi.org/10.1021/acs.chemrev.0c01174.
31. Mitronova, G. Y., V. N. Belov, …, S. W. Hell. 2010. New fluorinated rhodamines for optical microscopy and nanoscopy. *Chemistry*. 16:4477–4488. https://doi.org/10.1002/chem.200903272.
32. Alon, S., G. H. Huynh, and E. S. Boyden. 2019. Expansion microscopy: enabling single cell analysis in intact biological systems. *FEBS J.* 286:1482–1494. https://doi.org/10.1111/febs.14597.
33. Wassie, A. T., Y. Zhao, and E. S. Boyden. 2019. Expansion microscopy: principles and uses in biological research. *Nat. Methods*. 16:33–41. https://doi.org/10.1038/s41592-018-0219-4.
34. Trinks, N., S. Reinhard, …, U. Terpitz. 2021. Subdiffraction-resolution fluorescence imaging of immunological synapse formation between NK cells and A. fumigatus by expansion microscopy. *Commun. Biol.* 4:1151. https://doi.org/10.1038/s42003-021-02669-y.
35. Kunz, T. C., R. Götz, …, V. Kozjak-Pavlovic. 2020. Using Expansion Microscopy to Visualize and Characterize the Morphology of Mitochondrial Cristae. *Front. Cell Dev. Biol.* 8:617. https://doi.org/10.3389/fcell.2020.00617.
36. Truckenbrodt, S., and S. O. Rizzoli. 2021. Simple multi-color super-resolution by X10 microscopy. *Methods Cell Biol.* 161:33–56. https://doi.org/10.1016/bs.mcb.2020.04.016.
37. Truckenbrodt, S., C. Sommer, …, J. G. Danzl. 2019. A practical guide to optimization in X10 expansion microscopy. *Nat. Protoc.* 14:832–863. https://doi.org/10.1038/s41596-018-0117-3.
38. Truckenbrodt, S., M. Maidorn, …, S. O. Rizzoli. 2018. X10 expansion microscopy enables 25-nm resolution on conventional microscopes. *EMBO Rep.* 19:e45836. https://doi.org/10.15252/embr.201845836.
39. Lee, H., C.-C. Yu, …, P. Kosuri. 2021. Tetra-gel enables superior accuracy in combined super-resolution imaging and expansion microscopy. *Sci. Rep.* 11:16944. https://doi.org/10.1038/s41598-021-96258-y.
40. Nakamoto, M. L., C. Forró, …, B. Cui. 2022. Expansion Microscopy for Imaging the Cell–Material Interface. *ACS Nano*. 16:7559–7571. https://doi.org/10.1021/acsnano.1c11015.
41. Brinkmann, V., B. Laube, …, A. Zychlinsky. 2010. Neutrophil extracellular traps: how to generate and visualize them. *JoVE*. 36:1724. https://doi.org/10.3791/1724.
42. Chozinski, T. J., A. R. Halpern, …, J. C. Vaughan. 2016. Expansion microscopy with conventional antibodies and fluorescent proteins. *Nat. Methods*. 13:485–488. https://doi.org/10.1038/nmeth.3833.
43. Chen, F., P. W. Tillberg, and E. S. Boyden. 2015. Expansion microscopy. *Science*. 347:543–548. https://doi.org/10.1126/science.1260088.
44. Asano, S. M., R. Gao, …, E. S. Boyden. 2018. Expansion Microscopy: Protocols for Imaging Proteins and RNA in Cells and Tissues. *Curr. Protoc. Cell Biol.* 80:e56. https://doi.org/10.1002/cpcb.56.
45. Neubert, E., D. Meyer, …, L. Erpenbeck. 2020. The power from within – understanding the driving forces of neutrophil extracellular trap formation. *J. Cell Sci.* 133:jcs241075. https://doi.org/10.1242/jcs.241075.
46. Tachiwana, H., M. Dacher, …, N. Saitoh. 2021. Chromatin structure-dependent histone incorporation revealed by a genome-wide deposition assay. *Elife*. 10:e66290. https://doi.org/10.7554/eLife.66290.
47. Ma, B., T.-J. Trieu, …, X. Chen. 2020. Differential Histone Distribution Patterns in Induced Asymmetrically Dividing Mouse Embryonic Stem Cells. *Cell Rep.* 32:108003. https://doi.org/10.1016/j.celrep.2020.108003.
48. Zhu, Y., K. Gong, …, C. Murre. 2017. Comprehensive characterization of neutrophil genome topology. *Genes Dev.* 31:141–153. https://doi.org/10.1101/gad.293910.116.
49. Hergeth, S. P., and R. Schneider. 2015. The H1 linker histones: multifunctional proteins beyond the nucleosomal core particle. *EMBO Rep.* 16:1439–1453. https://doi.org/10.15252/embr.201540749.

Biophysical Reports, Volume *3*

Supplemental information

Expansion microscopy of neutrophil nuclear structure and extracellular traps

Jason Scott Holsapple, Lena Schnitzler, Louisa Rusch, Tobias Horst Baldeweg, Elsa Neubert, Sebastian Kruss, and Luise Erpenbeck

Supporting material

Expansion microscopy of neutrophil nuclear structure and extracellular traps

Jason Scott Holsapple[1*], Lena Schnitzler[2*], Louisa Rusch[3], Tobias Horst Baldeweg[3], Elsa Neubert[4], Sebastian Kruss[2,4,5+] and Luise Erpenbeck[1+]

1 Department of Dermatology, University Hospital Münster, Von-Esmarch-Strasse 58, 48149 Münster, Germany

2 Department of Chemistry, Ruhr-University Bochum, Universitätsstrasse 150, 44801 Bochum, Germany

3 Department of Dermatology, University Medical Center Göttingen, Robert-Koch-Strasse 40, 37075 Göttingen, Germany

4 Fraunhofer Institute for Microelectronic Circuits and Systems, Finkenstrasse 61, 47057 Duisburg, Germany

5 Center for Nanointegration Duisburg-Essen (CENIDE), Carl-Benz-Strasse 199, 47057 Duisburg

* Equal contribution

Corresponding authors: luise.erpenbeck@ukmuenster.de, sebastian.kruss@rub.de

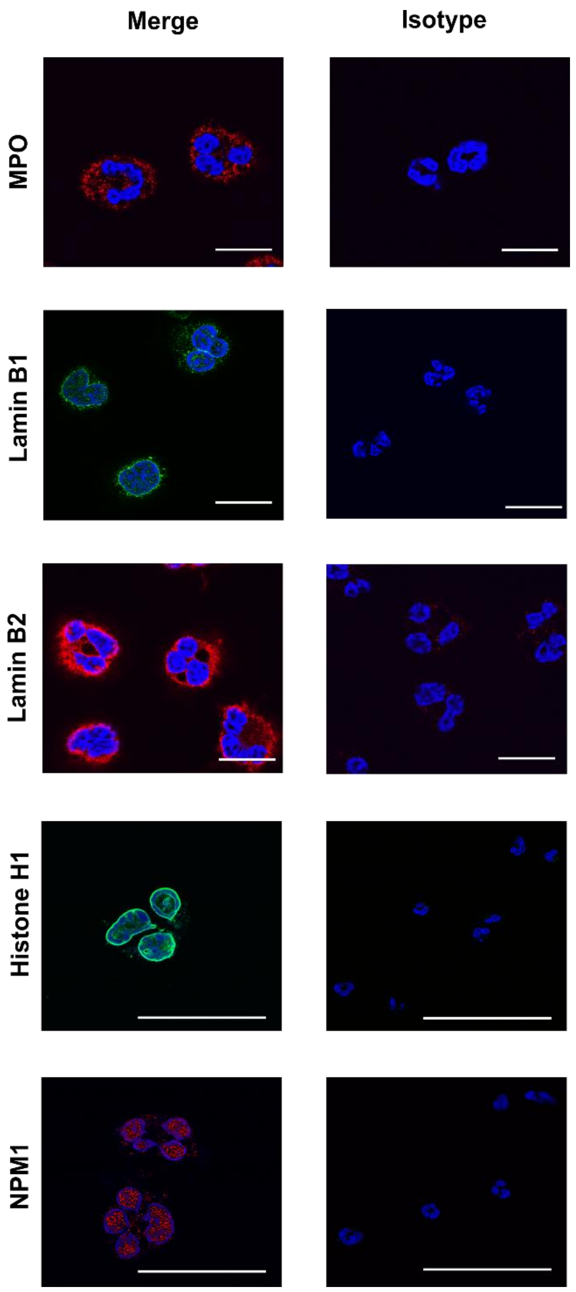

Supplementary Figure 1: Isotype controls for immunofluorescence staining of neutrophil structures, including cytoplasmic and nuclear proteins*: Myeloperoxidase (MPO, first row) as a cytoplasmic protein, Lamin B1 (second row) and Lamin B2 (third row) as components of the nuclear lamina of neutrophils, and histone H1 (fourth row) and nucleophosmin (NPM1, fifth row) as nuclear proteins. Column 1 shows expanded chromatin and stained structures merged. Column 2 shows the respective isotype controls for these structures. Scale bars = 50 µm.*

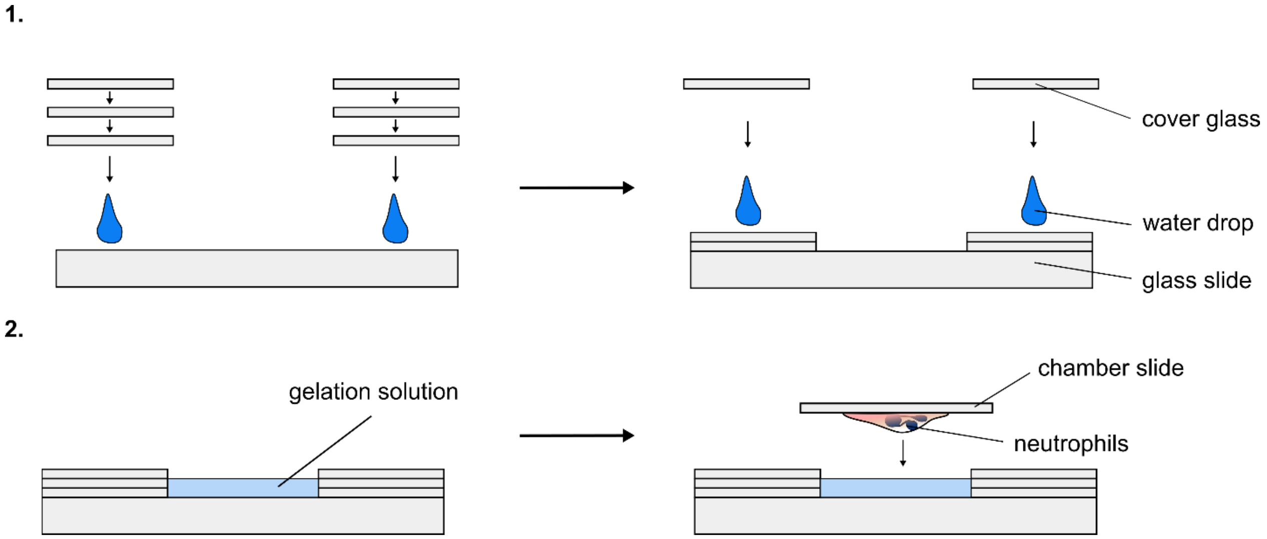

***Supplementary Figure 2: Schematic of gelation chamber assembly**. As outlined in the methods, a gelation chamber was assembled as an alternative to the 8-well chamber slides. Cover glass was attached to a glass slide using water drops, with a drop of water in between each layer of glass. The gelation solution was then introduced to the newly formed chamber and chamber slides with stained and labeled neutrophils were carefully lowered into the gelation solution.*

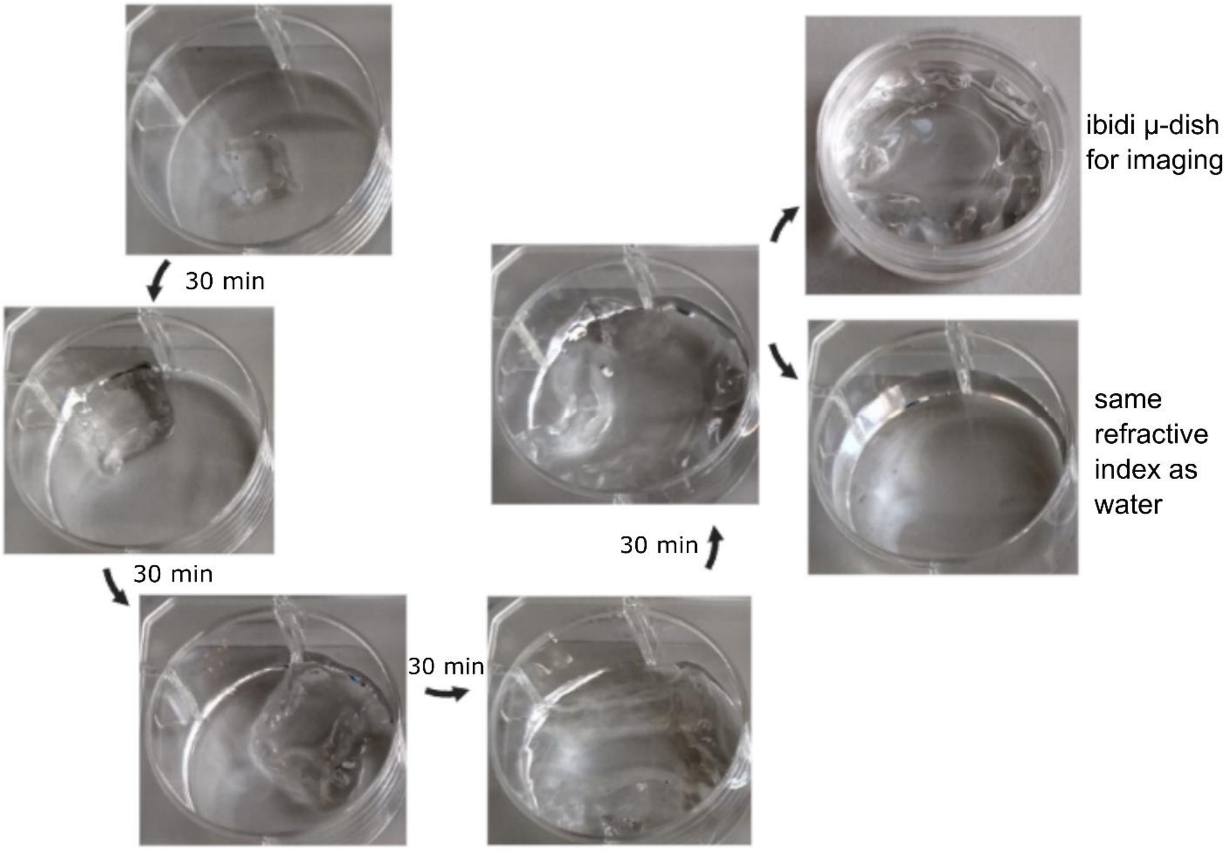

Supplementary Figure 3: Photographs of expansion time series. *Image series showing expansion of gel following addition and exchange of distilled water (30 minutes between each, pictures taken during water exchanges). An ibidi µ-dish was used for imaging, facilitating simple gel handling with higher objectives. Final image shows expanded gel in water, demonstrating the gel having matched the refractive index of water. Image created in BioRender.com.*

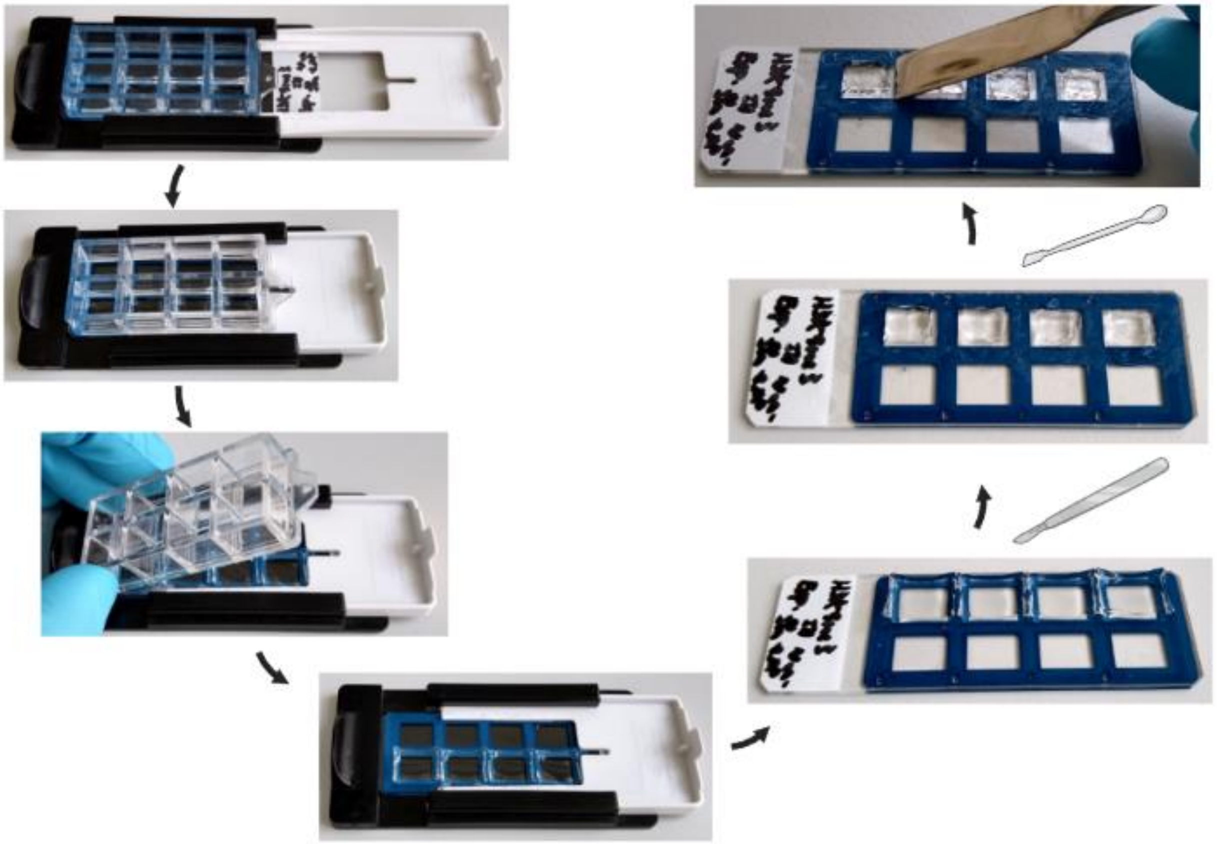

Supplementary Figure 4: Photographs of expansion microscopy technique (8-well format): *Example images of gel processing and removal from 8-well chamber slide (Nunc Lab Tek II). Following chamber removal, gel edges were removed using a disposable scalpel and then gels removed from the slide using a flat spatula. Image created in BioRender.com.*

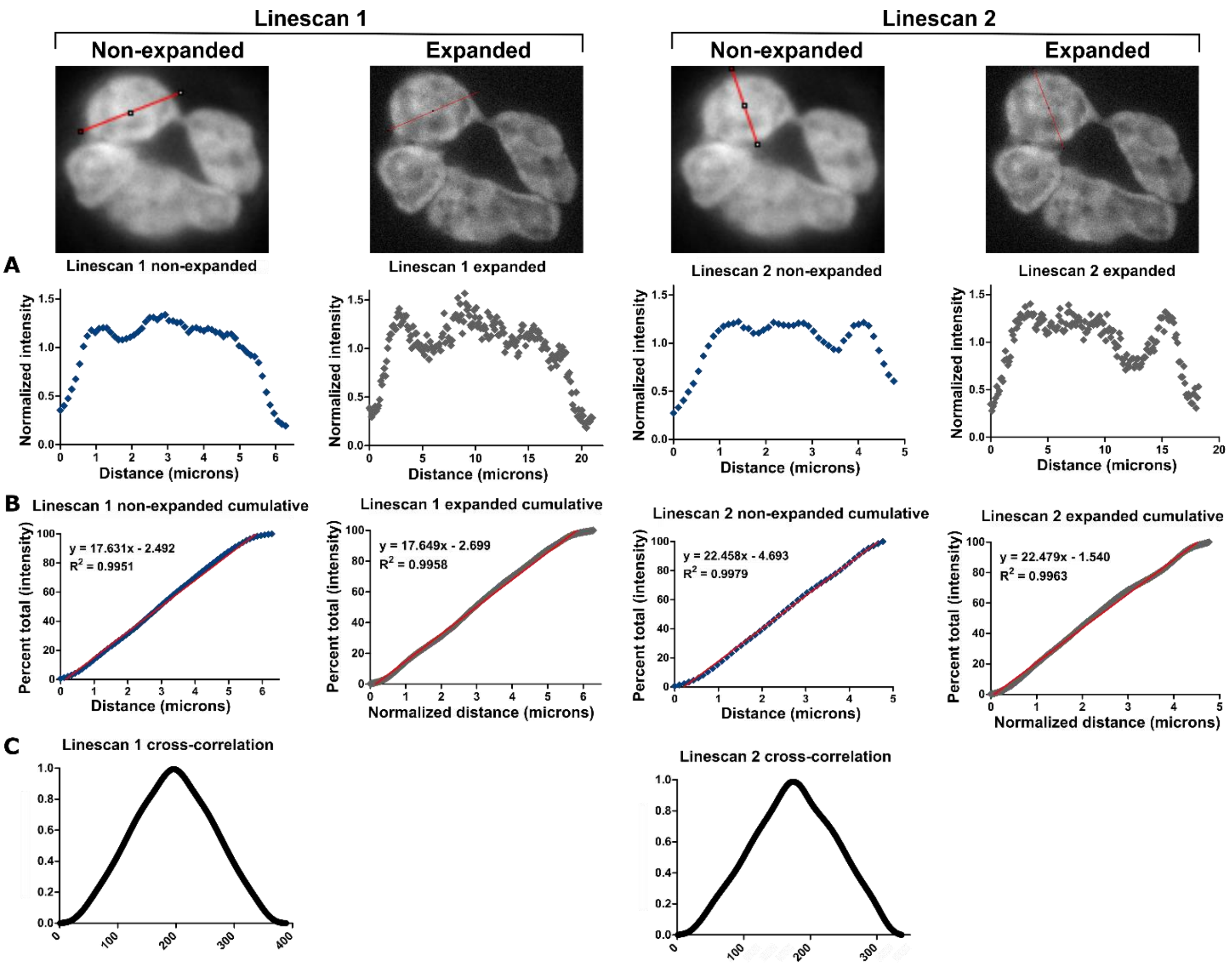

Supplementary Figure 5: Analysis of chromatin distribution pre- and post-expansion. *The distribution of chromatin in the same cell pre- and post-expansion was analyzed. The normalized intensity (gray value divided by mean gray value) corresponding to each line scan is shown in **A**. Cumulative plots representing the change in distribution of chromatin over the line scan are shown in **B**. Distance was normalized to the non-expanded range so the values could be compared, and a linear regression was performed. The high degree of similarity between the non-expanded and expanded slopes suggests similar distributions. Cross-correlation analyses of line scans from the non-expanded vs expanded cells are shown in **C,** and were performed using MATLAB (version R2019b) after data interpolation (linear method, 'interp1' function). The peak suggests a high degree of cross-correlation between the interpolated non-expanded and expanded line scans.*

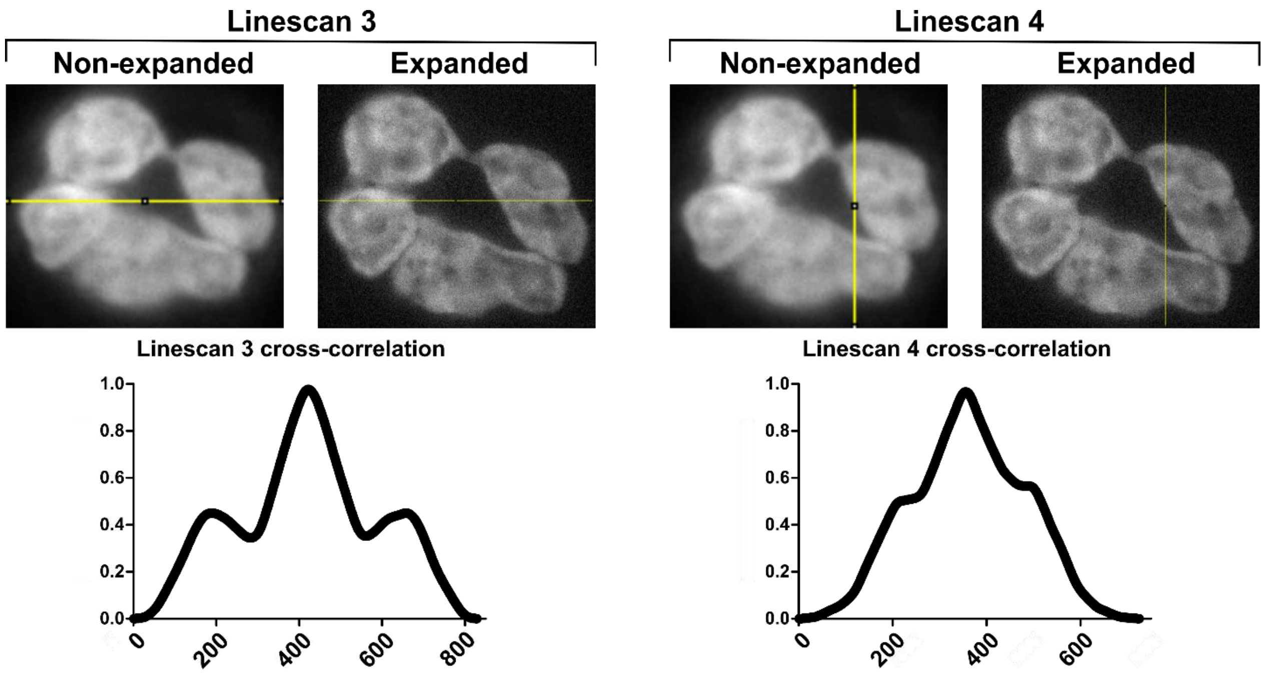

***Supplementary Figure 6: Cross-correlation analyses of whole-image line scans**. Additional line scans were subjected to cross-correlation analysis as in Figure 4. Whole image line scans were analyzed to further demonstrate the high degree of correlation between the same areas of the same cell pre- and post-expansion.*

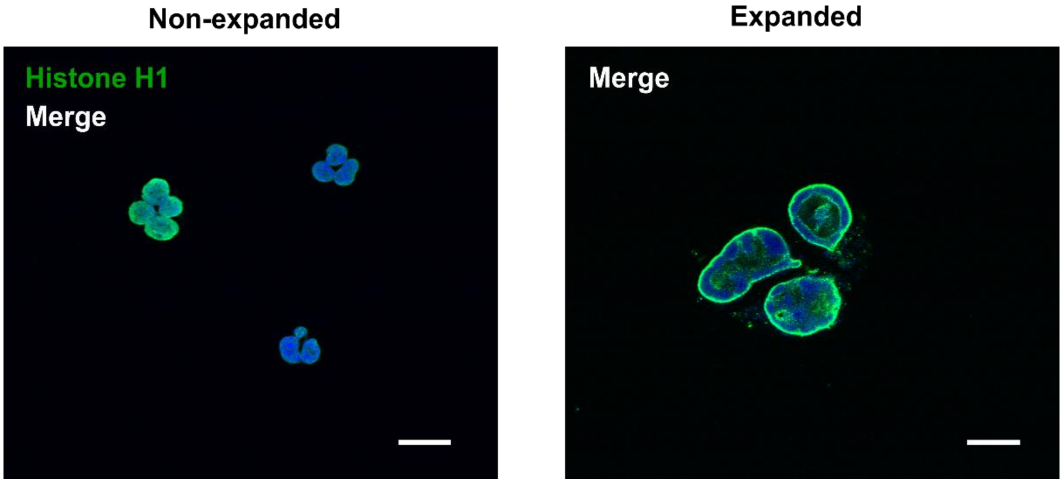

Supplementary Figure 7: Expansion microscopy and immunofluorescence staining of neutrophil nuclear border proteins*. Histone H1 was stained as a nuclear protein involved in unique higher-order structural conformations. Left shows non-expanded cells and right shows expanded cells. Scale bars = 10 µm.*

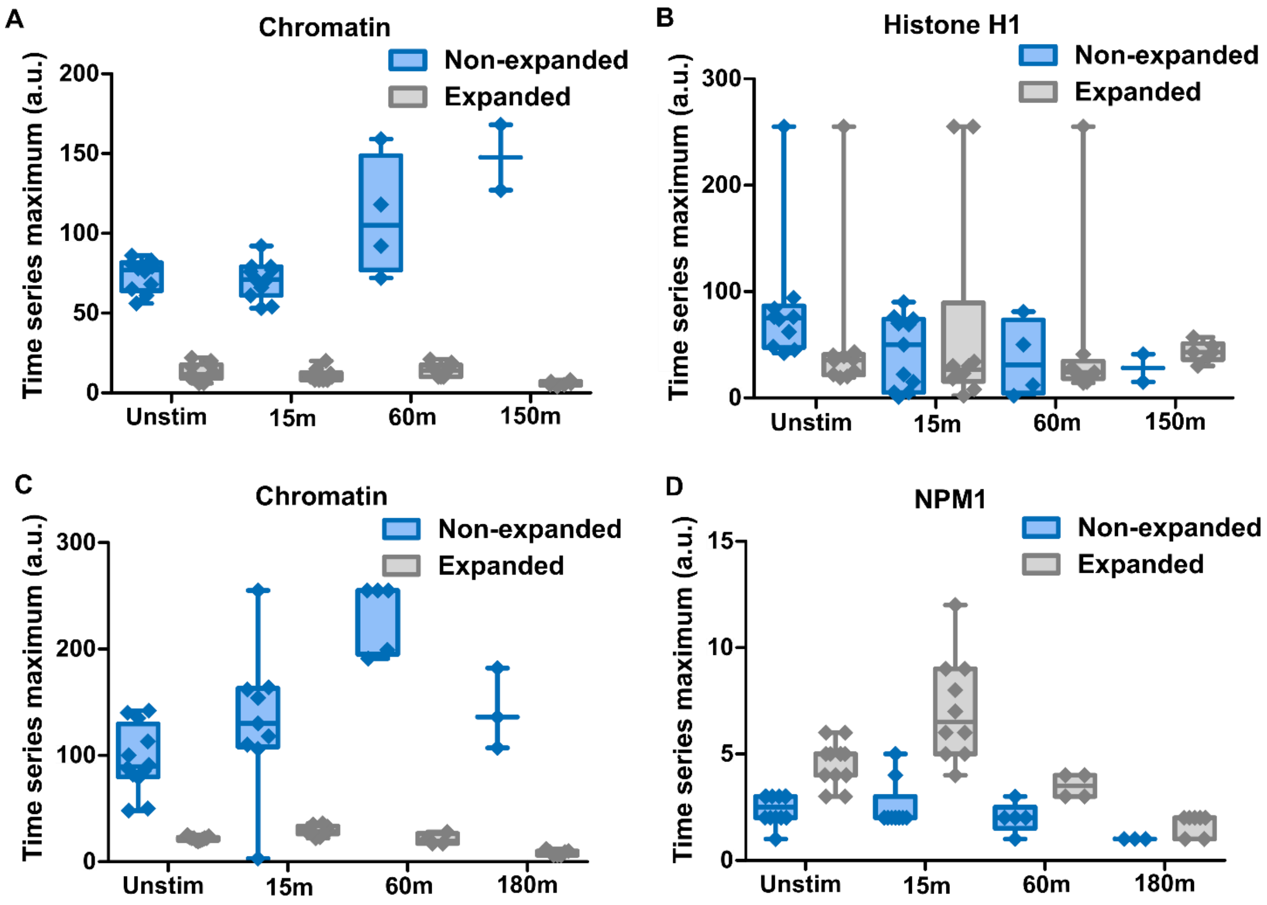

Supplementary Figure 8: Maximum intensity of paired chromatin and histone H1 or NPM1 in a time series*: Boxplots of the maximum intensity for chromatin and histone H1 (****A****,* ***B****) and chromatin and NPM1 (****C****,* ***D****). Plots are paired and respectively labelled as non-expanded and expanded for each. Whiskers represent the maximum and minimum for each boxplot.*

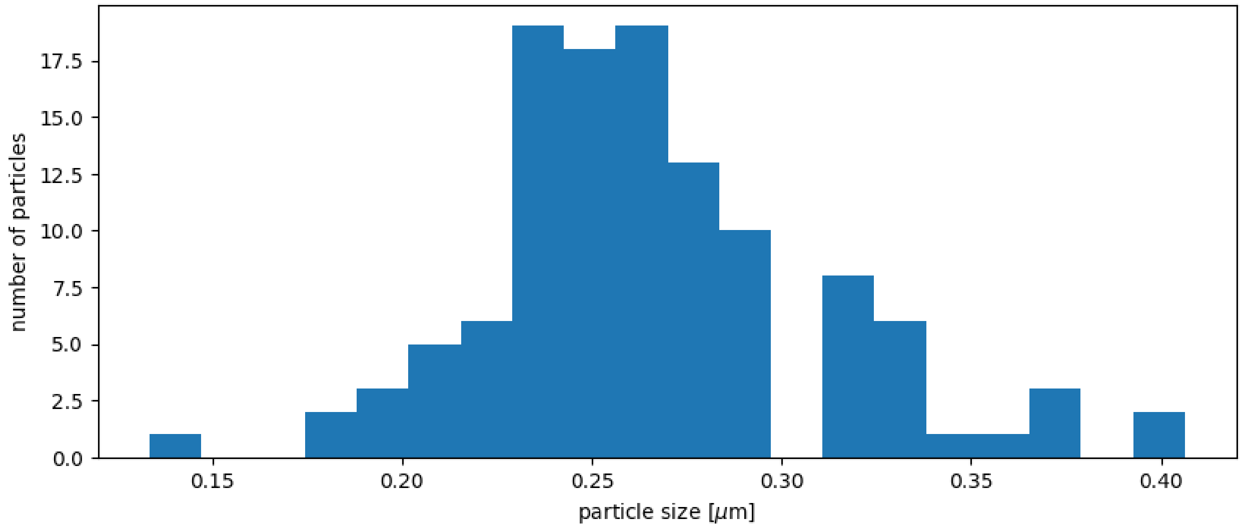

Supplementary Figure 9: Histogram of granular particle diameters*: The granular particle diameter of the histone H1 staining of a neutrophil after 150min is shown. The mean diameter is 266nm ± 45nm which corresponds to 54.29 nm ± 9.18 nm before expansion. The small granular structures are without expansion microscopy below the resolution limit and would not be visible.*

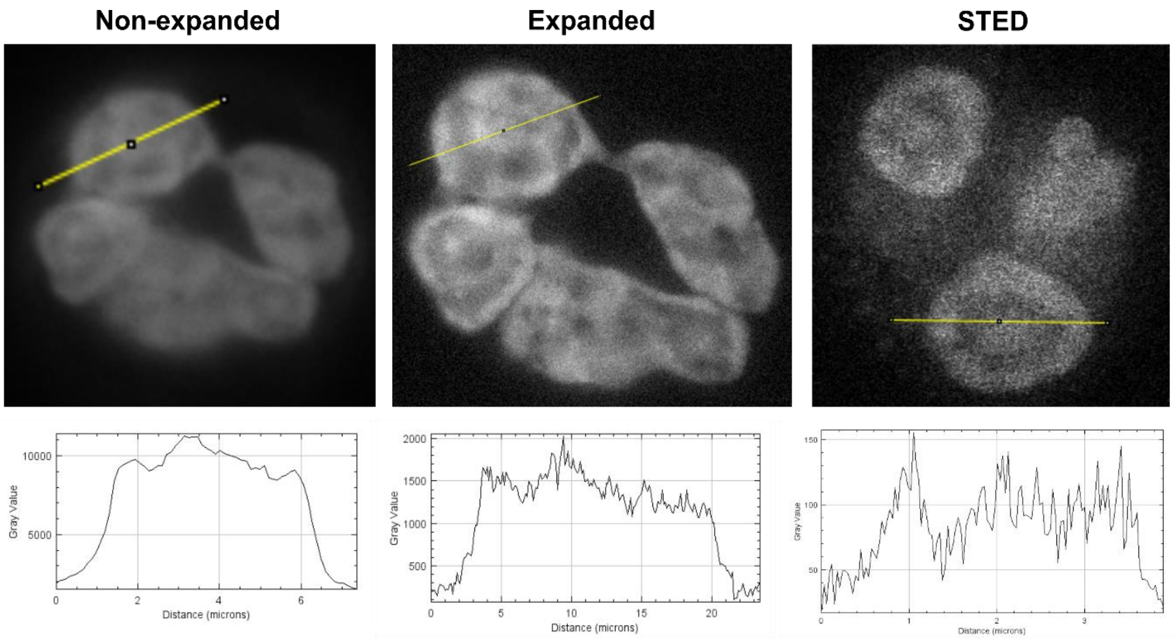

	Non-expanded	Expanded	STED
Percent maxima	11.59%	25.81%	31.30%

Supplementary Figure 10: Comparison of non-expanded, expanded, and STED percent maxima*. As a quantification of the level of detailed obtained in the above images, percent maxima was calculated by quantifying the number of peaks (individual local maximums corresponding to distinguishable points) and relating this to the total number of points measured by the respective line scan. There was a large gain in the level of detail with expansion, which was closer to the level observed with STED imaging. However, the level of detail obtained with STED is measured in typical (non-expanded) cells and so relatively this technique is still capable of a greater degree of super-resolution when strictly comparing to the gain in detail obtained with expansion.*

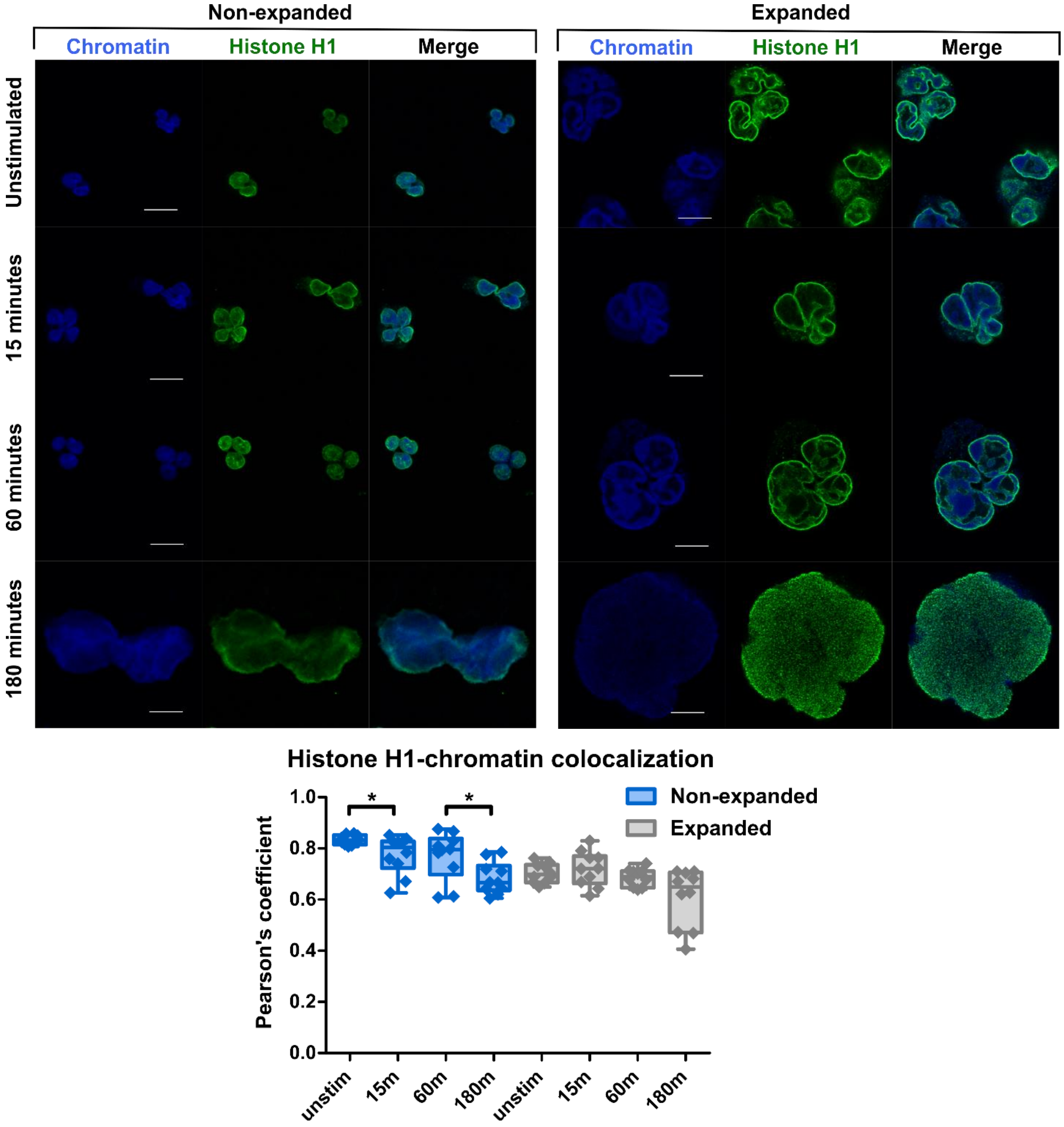

Supplementary Figure 11: Colocalization supplementary time series and measurements for histone H1 and chromatin*. Left shows non-expanded cells and right shows expanded cells. Graph is individual values (whiskers are minimum and maximum for each range); * = $p < 0.05$; n=10 cells analyzed per time point; images are PMA-stimulated human blood-derived neutrophils (100 nM), confocal images (63x); scale bars are 10 µm.*

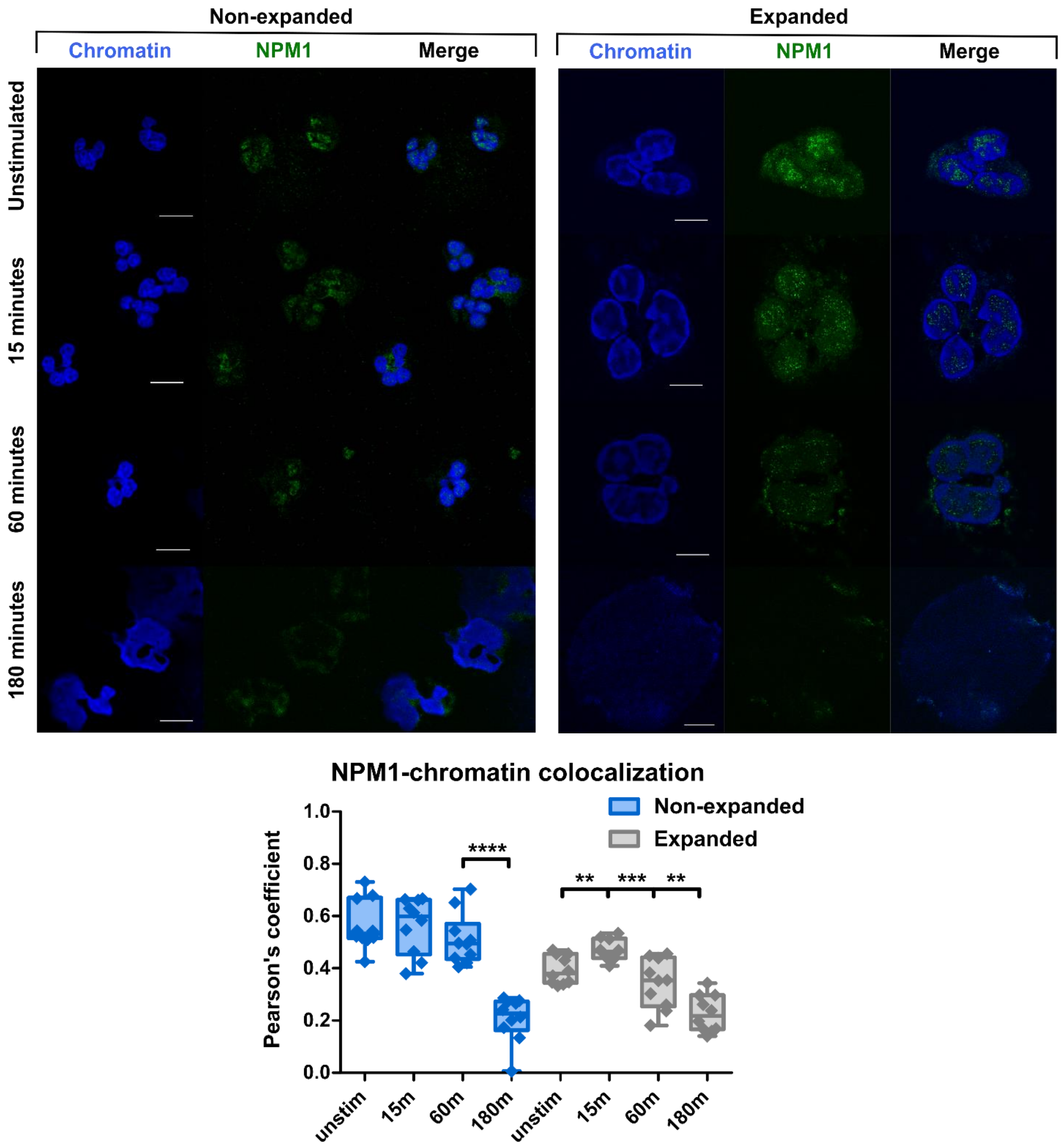

Supplementary Figure 12: Colocalization supplementary time series and measurements for NPM1 and chromatin*. Left shows non-expanded cells and right shows expanded cells. Graph is individual values (whiskers are minimum and maximum for each range); ** = p < 0.01, *** = p < 0.001, **** = p < 0.0001; n=10 cells analyzed per timepoint; images are PMA-stimulated human blood-derived neutrophils (100 nM), confocal images (63x); scale bars are 10 µm.*
